# Transcriptional Regulation of Pine Male and Female Cone Initiation and Development: Key Players Identified Through Comparative Transcriptomics

**DOI:** 10.3389/fgene.2022.815093

**Published:** 2022-03-18

**Authors:** Steffi Fritsche, Leonardo Rippel Salgado, Agnieszka K. Boron, Kyrin R. Hanning, Lloyd A. Donaldson, Glenn Thorlby

**Affiliations:** ^1^ Forest Genetics and Biotechnology, Scion, Rotorua, New Zealand; ^2^ Molecular and Digital Breeding, The New Zealand Institute for Plant and Food Research, Te Puke, New Zealand

**Keywords:** MADS-box, RNA-seq, gymnosperms, reproductive structures, cone development

## Abstract

With long reproductive timescales, large complex genomes, and a lack of reliable reference genomes, understanding gene function in conifers is extremely challenging. Consequently, our understanding of which genetic factors influence the development of reproductive structures (cones) in monoecious conifers remains limited. Genes with inferred roles in conifer reproduction have mostly been identified through homology and phylogenetic reconstruction with their angiosperm counterparts. We used RNA-sequencing to generate transcriptomes of the early morphological stages of cone development in the conifer species *Pinus densiflora* and used these to gain a deeper insight into the transcriptional changes during male and female cone development. Paired-end Illumina sequencing was used to generate transcriptomes from non-reproductive tissue and male and female cones at four time points with a total of 382.82 Gbp of data generated. After assembly and stringent filtering, a total of 37,164 transcripts were retrieved, of which a third were functionally annotated using the Mercator plant pipeline. Differentially expressed gene (DEG) analysis resulted in the identification of 172,092 DEGs in the nine tissue types. This, alongside GO gene enrichment analyses, pinpointed transcripts putatively involved in conifer reproductive structure development, including co-orthologs of several angiosperm flowering genes and several that have not been previously reported in conifers. This study provides a comprehensive transcriptome resource for male and early female cone development in the gymnosperm species *Pinus densiflora*. Characterisation of this resource has allowed the identification of potential key players and thus provides valuable insights into the molecular regulation of reproductive structure development in monoecious conifers.

## 1 Introduction

Planted forests cover less than 2% of global land yet play a crucial role in fulfilling the growing demand for industrial roundwood, thus reducing pressure on natural forests and providing social and environmental services (MacDicken et al., 2016). Conifers are the main tree type planted in commercial forests and the global production of industrial roundwood increased by 37% over the last 40 years (Brown and Ball, 2000; FOA, 2021). Wood sourced from plantation forests is expected to triple by 2050 in order to keep up with increasing demand (Payn et al., 2015). Failure to do so means that native forests will face a higher strain, a situation with disastrous consequences for biodiversity and climate mitigation efforts (WWF, 2015). Biotechnology offers tools to boost the productivity and sustainability of plantation forestry (Fenning and Gershenzon, 2002). Of particular interest is the control of reproduction of conifers via biotech tools. Trees without reproductive structures would facilitate the containment of genes introduced in genetically improved trees, reduce the amount of allergenic pollen, is predicted to increase growth and wood production while ensuring no unwanted wilding tree escapes into natural environments. Conversely, precocious flowering would help accelerating conventional tree breeding (Strauss et al., 1995; Fritsche et al., 2018; Roque et al., 2019).

Key challenges such as long generation intervals, complex highly repetitive genomes and the unavailability of reliable reference genomes for non-model species have made the investigations of genetic factors that control reproduction in conifers a slow process. However, several homologous genes thought to be involved in floral regulation in angiosperms have been identified in gymnosperms. These included genes from the MADS box transcription family which are part of the floral quartet or ABCDE model. This model is a general model for the specification of floral organs in angiosperms, which proposes that MADS-box proteins combine into different quaternary complexes that control floral organ identity (Theissen and Saedler, 2001; Rijpkema et al., 2010; Theißen and Gramzow, 2016). So far, orthologs of B, C/D and E-class floral homeotic genes and known to be involved in the control of meristem formation and organ identity have been identified in gymnosperms (Tandre et al., 1995, 1998; Mouradov et al., 1998; Rutledge et al., 1998; Sundström and Engström, 2002; Gramzow and Theissen, 2010; Melzer et al., 2010; Uddenberg et al., 2013; Gramzow et al., 2014; Silva et al., 2016; Chen et al., 2017). Other studies focusing on naturally occurring rare mutants such as early-cone setting or bisexual cone phenotypes, or using hormonal treatments to study genes expressed during reproductive structure development led to the identification of conifer-specific MADS-box genes such as the *DEFICIENS-AGAMOUS-LIKE* (*DAL*) genes (Niu et al., 2015; Zhao et al., 2015; Feng et al., 2018).

To achieve both male and female sterility, an intervention at an early stage of the flowering process is necessary. But the inability to predict which of the large number of vegetative meristems in conifers will transition from vegetative to reproductive organs has made the research into initial and early development of reproductive structures a difficult undertaking. In this study, we utilise the highly floriferous species, *Pinus densiflora* to investigate cone initiation and development. This species, naturally distributed throughout Japan, Korea and parts of Russia and China, reproduced in our New Zealand based nursery at a young age and a large proportion of the vegetative meristems transition to reproductive development. Thus, the coning on almost all branches makes *P. densiflora* a perfect species to study reproductive structure development. We investigated the transcriptomes of buds and male and female cones at four different time points by comparative, phylogenetic and GO enrichment analyses. We scrutinized genes that we found annotated to flower related GO terms and were differentially expressed in our samples. Finally, we show and discuss the relationships and expression patterns of *P. densiflora* MADS-box type II genes in the developing cones. With these findings this study presents new insights into the regulation of early cone initiation and development in conifers and provides potential candidates to advance sterile or early flowering conifer trees.

## 2 Materials and Methods

### 2.1 Sample Collection

Two year old seedlings of Japanese red pine (*P. densiflora* Sieb.& Zucc.) were obtained from Appletons nursery in June 2011 and were grown in 25 L pots at SCION, Rotorua, New Zealand (38°09′31.3*″*S, 176°16′08.2*″*E, elevation 321 m). Samples were collected from three individual trees during August to mid October 2016 (Table 1) which covers the early reproductive structure development from cone emergence to pollen shedding (Supplementary Figure S1). Pine cone development has been described elsewhere (Williams, 2009). In short, *P. densiflora* male structures developed in a cluster at the base of lateral shoots, circa two to 3 weeks before the female cones (Supplementary Figure S1) (Goo, 1961). Female structures emerged individually or in pairs at the tips of buds. To cover early female cone development, the apex of a bud, sampled at the earliest time point, was isolated by cutting off the first 5 mm of the tip (apex) of the bud, thus including any female or vegetative primordia but excluding any possible male reproductive structures. In addition, needles and buds from lateral shoots with no visible reproductive structures were sampled at the first sampling time point (Table 1). All samples were snap-frozen in liquid nitrogen and stored at −80°C until used. For RNA isolation, male and female reproductive structures (MC-1 to MC-3/FC-1 to FC-3) were separated from the bud (Table 1).

**TABLE 1:** *P. densiflora* tissue collections. MC: male cones, FC: females cones.

Sample name	Sample description	Sampling time	Number of trees
Needles	Needles	30. August 2016	3
Buds	Buds	30. August 2016	3
MC-1	Male cone small (<2 mm)	06. September- 13. September 2016	3
MC-2	Male cone medium (<3 mm)	26. September- 07. October 2016	3
MC-3	Male cone mature (<5 mm)	14. October- 21. October 2016	3
Apex	Lateral bud apex	30. August- 13. September 2016	3
FC-1	Pre-open (>2 mm)	26. September 2016	3
FC-2	Female cone partial open	3. October- 07. October 2016	3
FC-3	Female cone fully open	07. October- 14. October 2016	3

### 2.2 Microscopy of *P. densiflora* Reproductive Structures

*P. densiflora* samples were fixed and stored in FAA solutions (5% formaldehyde, 5% acetic acid, 90% ethanol). Samples were washed in ethanol to remove the fixative and to dehydrate the sample before infiltration in LR White resin for several weeks. Blocks containing individual buds were polymerised for 2 days at 65°C and subsequently polished with a range of abrasive papers (320–400 grit) using a Mecapol P230 grinding unit (Presi, Grenoble, France), to produce a medial longitudinal surface as described in Dickson et al. (2017). The embedded block was then imaged using confocal microscopy (Leica SP5 II, Leica Microsystems, Wetzlar, Germany) by detecting the natural auto-fluorescence of the cells. Individual maximum intensity projections were used to visualise bud development. Excitation was 488 and 561 nm with 500–550 nm and 570–700 nm emission respectively using a 20x objective lens and 1024 × 1024 or 2048 × 2048 pixel resolution. Male cone development was observed by evaluating the presence of microsporangia, microspores and pollen grains; and female cone development by observing the presence of ovule development.

### 2.3 RNA Extraction and Quality Assay

All samples were cryogenically pulverized in the Geno/Grinder® 2000 (SPEX CertiPrep™, Rickmansworth, United Kingdom) at 1,500 rpm for 2 min and using 3 mm × 3 mm metal balls per sample. Total RNA from the samples was extracted using the Spectrum™ Plant Total RNA Kit (Sigma-Aldrich/Merck KGaA, Darmstadt, Germany) following the manufacturer’s instructions. Subsequently, the samples were subjected to ethanol precipitation. The total RNA quantity and purity was assessed by using the Qubit RNA Assay Kit (Invitrogen™, Waltham, United States) as well as using Bioanalyzer 2,100 (Agilent, Santa Clara, United States).

### 2.4 Droplet Digital PCR and Data Analysis

Following the manufacturer’s protocol of the iScript gDNA Clear cDNA Synthesis Kit (Bio-Rad Laboratories, Auckland, NZ), 1 *μ*g total RNA for each of the nine samples (buds, apex, FC-1, FC-2, FC-3, MC-1, MC-2, MC-3, and needles) and three biological replicates was used to generate cDNA. All cDNAs were normalised to 2.5 ng μl^−1^ prior to droplet digital PCR (ddPCR). Technical replicates of the RT step were completed to assess RT efficiency. Transcript names and oligonucleotides used in ddPCR are listed in Supplementary File S1. The Bio-Rad C1000 and QX200 systems (Bio-Rad Laboratories, Auckland, NZ) were used to perform the ddPCR according to the manufacturer’s instructions and as previously described (Hindson et al., 2011). Then ddPCR data was analysed using the 102 QuantaSoft software (Bio-Rad, Auckland, NZ) following the manufacturer’s recommendation. Wells with < 10,000 accepted droplets were excluded from analysis. The fluorescence threshold was set at approximately one standard deviation above the droplets in the control wells (no template). Target concentrations in copy number µl reactions were automatically calculated by QuantaSoft.

### 2.5 Transcriptome Sequencing

Illumina sequencing libraries were prepared by Otago Genomics & Bioinformatics facility using a total of 500 ng RNA/library with the TruSeq stranded mRNA sample preparation kit (Illumina, NY, United States) as per the manufacturer’s guidelines. In short, polyA containing mRNA molecules were captured using poly-T oligos and then fragmented. First strand cDNA synthesis of the cleaved RNA fragment was done by random hexamer priming and reactions include actinomycin D to prevent DNA-dependent synthesis and to improve strand specificity. Strand specificity was achieved by replacing dTTP with dUTP during second strand synthesis, quenching the second strand during amplification with polymerase I and RNase H. A single “A” base was added to the resulting cDNA fragments ready for adapter ligation. Proceeding with ligation, the resulting library fragments were purified and enriched using 15 cycles of PCR to create the final cDNA libraries. An equimolar pool of 27 libraries was assessed on the Illumina MiSeq, prior to loading onto the Illumina HiSeq2500 sequencer.

### 2.6 Transcriptome Assembly, Assessment and Annotation

FastQC (S. Andrews: http://www.bioinformatics.bbsrc.ac.uk/projects/fastqc) was used to check general quality of the raw reads. Subsequently, the reads were processed with trimmomatic (Bolger et al., 2014) using a sliding window approach (SLIDINGWINDOW:4:20) and removing adapter contamination (TruSeq3-PE.fa:2:30:10:2). After trimming, reads shorter than 150 base pairs were removed, representing a more conservative setting for read combination. This allowed more than 80% of all reads to be combined in almost all libraries. Each of the libraries was assembled using Trinity (Grabherr et al., 2011) using Kmer settings of 25 and 31 and otherwise standard settings. A genome guided assembly was carried out using Trinity De novo Transcriptome Assembly Genome Guided pipeline (Grabherr et al., 2011) with default parameters and using the *Pinus taeda* genome from the Gymno PLAZA version 1.0 website as reference. All the assemblies were then combined into one file and filtered using the EvidentialGene pipeline (Gilbert, 2019) to obtain best isoforms from the various assemblies. This final data set was classified and annotated using Transcriptome Functional Annotation and Analysis (Trinotate) (Bryant et al., 2017). Trinotate is a suite for functional annotation of transcriptomes, it uses a number of different well referenced methods for functional annotation, including homology search against sequence databases (BLAST+/SwissProt), protein domain identification (HMMER/PFAM), and comparison to currently curated annotation databases (eggNOG, Gene Ontology terms). The list of references for each individual programs used by Trinotate pipeline can be found on https://github.com/Trinotate/Trinotate.github.io/blob/master/index.asciidoc.

### 2.7 Comparison to Other RNA Assemblies

To assess the assembled transcriptome completeness, the proteome shotgun assemblies for *Picea abies, P. pinaster, P. sylvestris*, and *P. taeda* from the Gymno PLAZA 1.0 website (https://bioinformatics.psb.ugent.be/plaza/versions/gymno-plaza/download/index) and functional comparison of annotated sequence data was performed using the Mercator tool (Mercator4 v2.0) (Schwacke et al., 2019).

### 2.8 Gene Ontology, Read Mapping and Differential Expression Analysis

To enable multiple comparisons between samples and time points, a single Trinity assembly for each of the samples were generate followed by an abundance estimation using the *align_and_estimate_abundance.pl* script from the Trinity package concomitant with the Salmon transcript quantification tool (Patro et al., 2017). Furthermore, the transcript abundance estimates for each sample were used to obtain a global matrix of counts and normalised expression values by using the *abundance_estimates_to_matrix.pl* from trinity package and the trimmed mean of M values (TMM) option for normalisation. Then, a filtering step was performed retaining transcripts that had an estimated read-count of 16 in at least three samples each. Given the sequencing depth and that each biological sample was represented by three replicates, this represented a relatively modest filtering of about 0.5 counts per million in at least one sample. This expression based additional filter reduced the number to 37,164 transcripts in total used for downstream expression analysis.

To investigate relationships among biological samples, the correlation of biological replicates was investigated using the fragment counts matrix generated by abundance estimation, with no filtering, as input followed by a counts-per-million (CPM) data transformation followed by a log2 transform performed by the *PtR* script of Trinity package.

Subsequently, differential expression analysis was performed using the matrix of non normalised counts as input for DESeq2 (Love et al., 2014) by *run_DE_analysis.pl* script from Trinity package, modelling both, tissue types and time points, as separate factors. Differential expression was determined using the DESeq2 dispersion method and by using a Wald-test with the female developmental stages (FC-1, FC-2, FC-3), apex, as well as the male developmental stages (MC-1, MC-2, MC-3) and the bud stage.

Gene Ontology enrichment analysis of differentially expressed transcripts for all pairwise samples was conducted on the GO annotations previously acquired by Trinotate using the *extract_GO_assignments_from_Trinotate_xls.p* script and GOseq R package (Young et al., 2010) using the *run_GOseq.pl* script, both from Trinity package. Normalised counts from the transcripts annotated with floral related GO terms, obtained from the enriched libraries, were used for plotting a word-cloud panel using the R package ggplot2 (Wickham, 2011).

The R packages “venn” and “UpSetR” (Conway et al., 2017; Dusa, 2021) were used for visualisation of the dataset composition. The results of the pairwise DEG analysis and Gene Ontology enrichment analysis are available in Supplementary File S4.

### 2.9 Phylogenetic and Expression Analysis of MADS-Box Genes

To verify the genetic relationships shared with other species and sequence conservation of MADS-box genes from *P. densiflora*, amino acid sequences from *Picea abies* and *P. tabuliformis* were selected from PSI-BLAST alignment using default parameters (Altschul et al., 1997). Only full-length protein sequences that contained a K-box domain and a SRF-domain within the coding sequence, identified manually from PSI-BLAST results, were used. Then, T-COFFEE Version_13.39.0.d675aed (Notredame et al., 2000) in accurate mode was used to perform the alignment. The fasta result of the alignment was used as input to RAxML (Randomized Accelerated Maximum Likelihood) (Stamatakis, 2014), with 1,000 bootstraps to reconstruct the phylogenetic relationships between MADS-box sequences. The generated phylogenetic tree was visualized using iTOL (Interactive Tree Of Life) v5 (Letunic and Bork, 2019). To assign the 54 *P. densiflora* sequences into gene families and assess their relationship to *Picea abies* and *P. tabuliformis* genes, a phylogenetic analysis was conducted. To do so, first, the 54 amino acid sequences contained in a single fasta files were used as a query for a PSI-BLAST (Altschul et al., 1997) using the NR-Protein database and with the pre-selected organisms (*Arabidopsis thaliana, Picea abies* and *P. tabuliformis*) and default parameters. Then, the first PSI-BLAST top hits were used for a protein alignment and constructing the phylogenetic tree. For tree rooting, the floral meristem identity gene *FLORICAULA/LEAFY* was used, as studies have shown that it shares the same ancestry as MIKC-type MADS box genes (Moyroud et al., 2017; Gao et al., 2019).

The expression levels (DESeq2 normalised counts) of *P. densiflora* MADS-box type II genes were used for an expression pattern analysis using hierarchical clustering and heatmap visualisation. The heatmap was generated using the pheatmap package for R and expression levels were transformed using the “rlog” function to adjust for differences in library sizes. Transcripts were hierarchically clustered based on relation to time-points and tissue types.

## 3 Results

### 3.1 Morphological Observation of *P. densiflora* Reproductive Structures at Different Time Points

**FIGURE 1:**
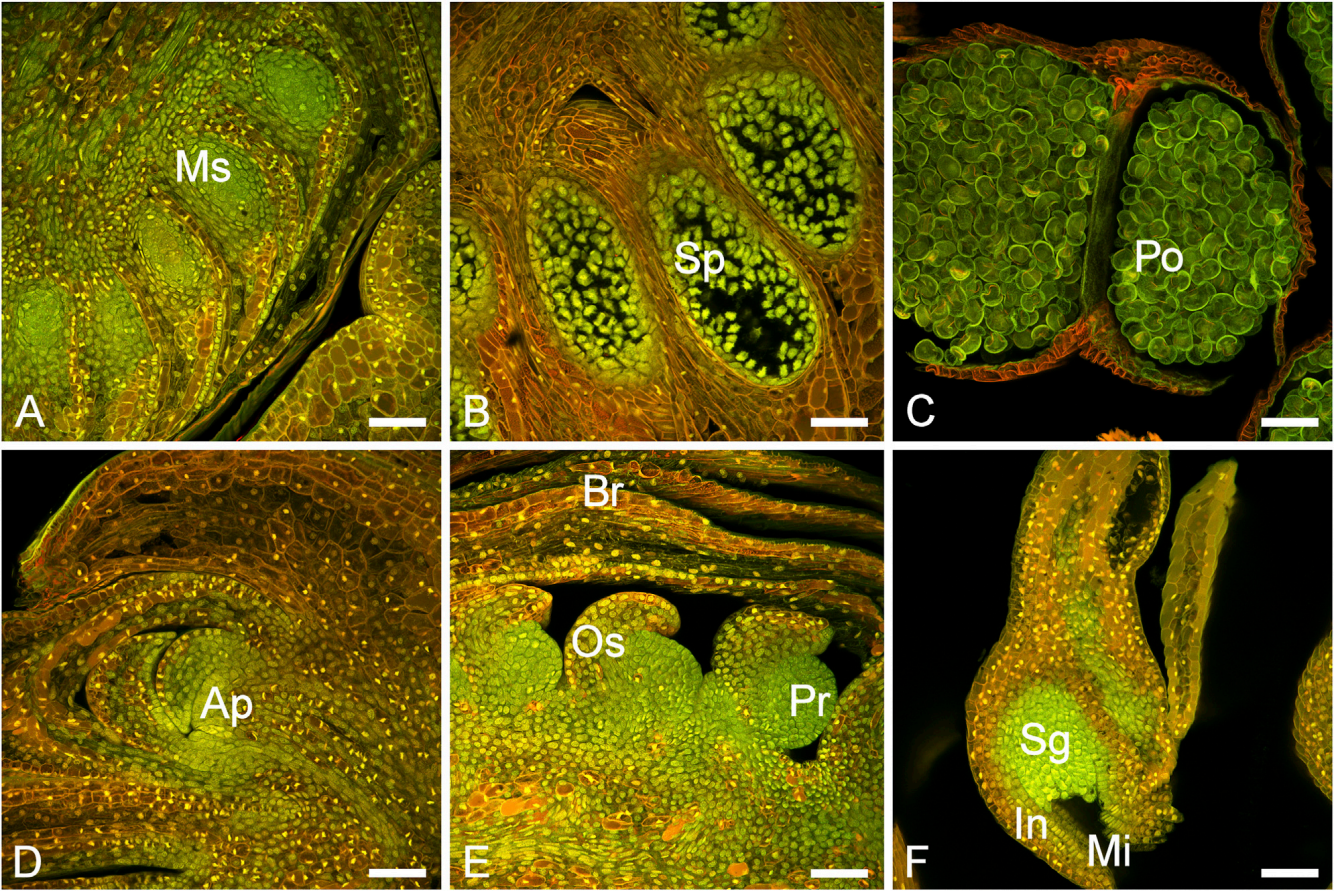
Male and female cone development in *P. densiflora*. **(A)** Immature male bud (MC-1) with developing microsporangia (Ms). **(B)** Immature male cone (MC-2) with sporocytes (Sp). **(C)** Mature male cone (MC-3) with pollen grains (Po). **(D)** Vegetative bud (apex) showing the apical meristem (Ap). **(E)** Developing female bud (FC-1/FC-2) showing ovuliferous scales (Os), primordium (Pr) and bracts **(B)**. **(F)** A mature ovule (FC-3) showing the sporangium (Sg), integuments (In) and micropyle (Mi). Scale bars = 100 nm.

The *P. densiflora* male structures developed circa 10–20 days earlier than the female structures and in a cluster in the proximal part of the shoots (Supplementary Figure S1). During the following 8 weeks, the male cones enlarged, eventually turned from green to yellow and released pollen (Supplementary Figure S1). Female structures emerged on the distal part of the shoots and were usually growing on branches in the upper third of the trees (Supplementary Figure S1). Shoots and cones were inspected by microscopy throughout the sampling. Developing microsporangia and ovules could be observed (Figure 1). The longitudinal section of shoots sampled at the earliest time point (“buds”) revealed the beginning of very early immature male cone development.

### 3.2 Sequencing and Assembly of Transcriptomes

Illumina sequencing was used to analyse the transcriptional profiles during cone development of the samples. This generated 1,531,306,169, pair-end, 150 bp long reads representing 382.82 Gbp with an average of ≥ Q30 of 92%. To obtain the best possible assembly, we combined all assemblies, *de novo* and genome guided, into one large redundant file. Subsequently, assembly isoforms were filtered out using the EvidentialGene Pipeline (Gilbert, 2019). The resulting dataset contained 150,661 genes and 157,328 transcripts.

### 3.3 Transcriptome Annotation and Functional Characterisation

We functionally annotated and compared our dataset containing 150,661 genes and 157,328 transcripts with publicly available genomic resources of *P. taeda, P. pinaster, Picea abies*, and *P. sylvestris* from PLAZA 1.0 (Uddenberg et al., 2013) using the Mercator plant pipeline (Schwacke et al., 2019). In total, 19.48% of the transcriptome, represented by 12,172 transcripts, could be classified into functional bins and 32.33% of transcripts could be functionally annotated. Based on the putative functional classification of our predicted proteins, we could assign them to their respective MapMan bins. Using this approach, we could designate at least one transcript to 4,182 of 4,500 MapMan bins/classes. From those, we identified 87 MapMan bins that had at least one representative in the *P. densiflora* transcriptome assembly but not in any of the other conifer transcriptome assemblies. And conversely, there were 1,193 categories found in at least one of the other Pinaceae members assemblies but not in *P. densiflora* (Supplementary Figure S2). The full annotation results by Mercator tool and Trinotate are available as Supplementary Files S2, S3.

### 3.4 Differential Gene Expression of Male and Female Cones

Pairwise differential expression analysis of all tissues and time points (Supplementary File S4) identified 172,092 differentially expressed genes (DEGs) in overall 36 pairwise comparisons, regardless of LogFC cutoff (Table 2). The highest number of DEGs was observed when comparing needles vs. all other samples, up to 13,655 DEGs. There were only small differences in the number of DEGs between the apex and female cone samples. The pairwise comparison of MC-1 vs. MC-2 had the lowest numbers of DEGs (838) and MC-1 vs. MC-3 with 5,900 DEGs the highest number.

**TABLE 2:** Differentially expressed transcripts comparison between samples. Cutoff Log2 fold change ≥ 2 and ≤ (−2), FDR 0.05, Apex, FC: female cones, MC: male cones.

	Bud	Apex	FC-1	FC-2	FC-3	MC-1	MC-2	MC-3	Needles
Bud	0	656	1,043	1,448	1903	1,513	3,834	7,726	10,058
Apex	656	0	250	791	1,318	1,651	3,326	5,899	9,902
FC-1	1,043	250	0	177	830	5,701	4,422	6,468	13,655
FC-2	1,448	791	177	0	48	3,888	3,825	7,103	12,712
FC-3	1903	1,318	830	48	0	2,282	3,457	6,821	11,622
MC-1	1,513	1,651	5,701	3,888	2,282	0	838	5,900	9,639
MC-2	3,834	3,326	4,422	3,825	3,457	838	0	4,500	9,001
MC-3	7,726	5,899	6,468	7,103	6,821	5,900	4,500	0	7,885
Needles	10,058	9,902	13,655	12,712	11,622	9,639	9,001	7,885	0

**FIGURE 2:**
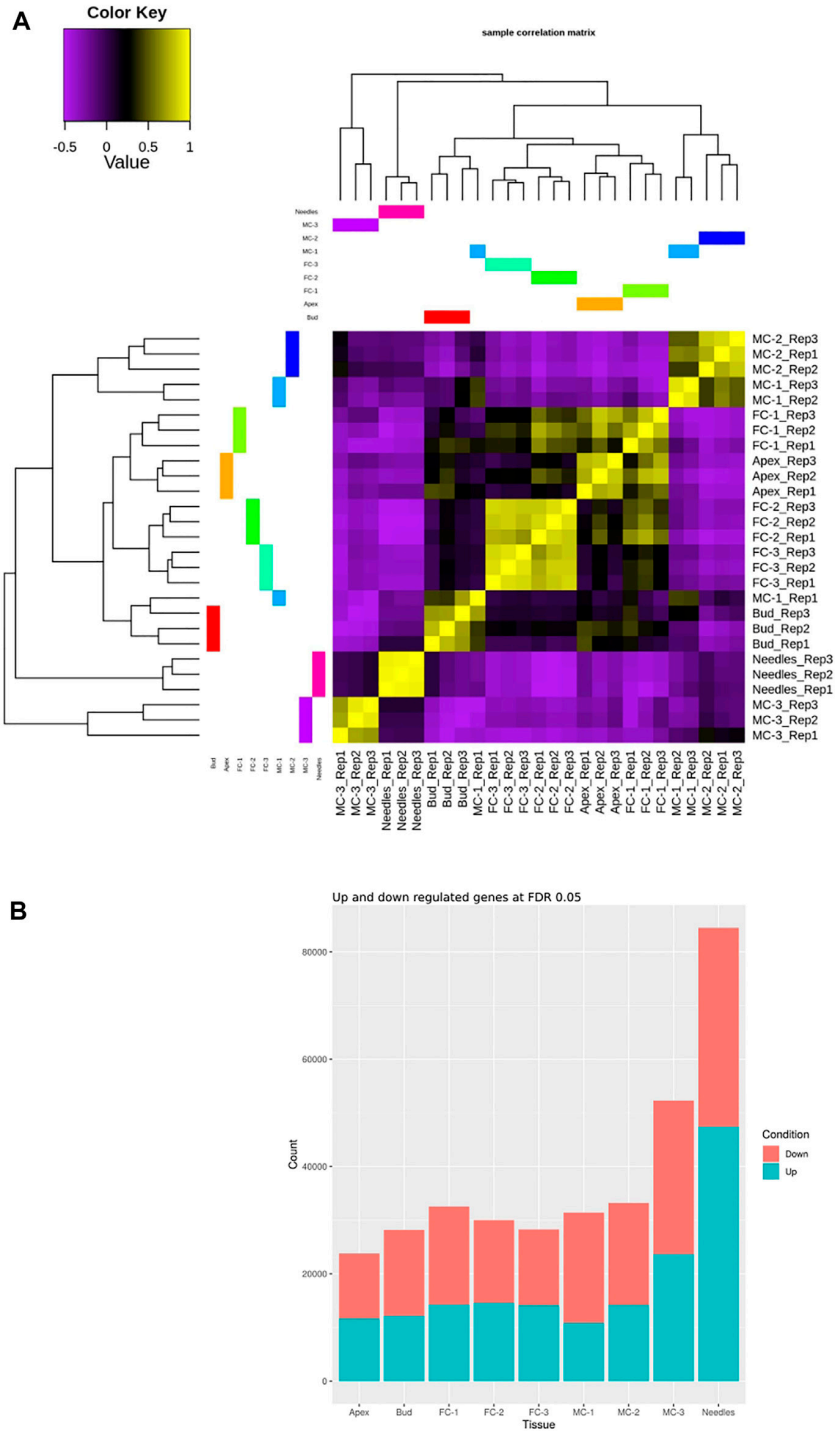
Comparison of the transcriptome relationships and DEGs of the examined samples. **(A)** Correlation matrix of the transcriptome libraries of five tissues and four time points (needles, buds, apex, MC-1, MC-2, MC-3, FC-1, FC-2, FC-3). **(B)** Sum of identified DEGs for each sample individually compared against all tissues and time points for a cutoff of logFC ≥2 and ≤ ( − 2), and FDR 0.05. The Needle tissue have more DEGs in comparison to any other tissue sampled.

Clustering analysis revealed a good distinction between tissue types and a strong correlation (sample correlation coefficient between 0.75 and 1) among the replicates with one outlier (MC-1-replicate 1) (Figure 2A). The transcriptomes of the female cone samples collected during later developmental time points (FC-2, FC-3) were highly similar to each other (sample correlation coefficient between 0.75 and 1). MC-1 and MC-2 clustered closer together, while the last male cone time point (MC-3) separated distinctly from the other male cone transcriptomes. The transcriptomes of needles clustered more closely but were still clearly separated from the MC-3 transcriptome, while bud and apex samples formed a sub-cluster with all female transcriptomes. Overall, the dendrogram clustered samples according to temporal dynamics and organ identity (Figure 2A).

During female cone development the sum of up-regulated genes found in all pairwise comparisons increased slightly from circa 43% in FC-1, to 50% in FC-3 (Figure 2B). In male cone samples the total number of up-regulated genes per sample was lower but also increased, from 37% to about 43% during development (Figure 2B). To verify the findings of our expression analysis, we randomly selected nine genes and analysed their expression using droplet digital PCR (ddPCR). We found analogous expression patterns in the individual tissue types when comparing ddPCR results with RNA-seq data of the corresponding gene. This confirmed correct measures of transcript levels from our RNA-seq data on transcript expression level (Supplementary Figure S3).

**FIGURE 3:**
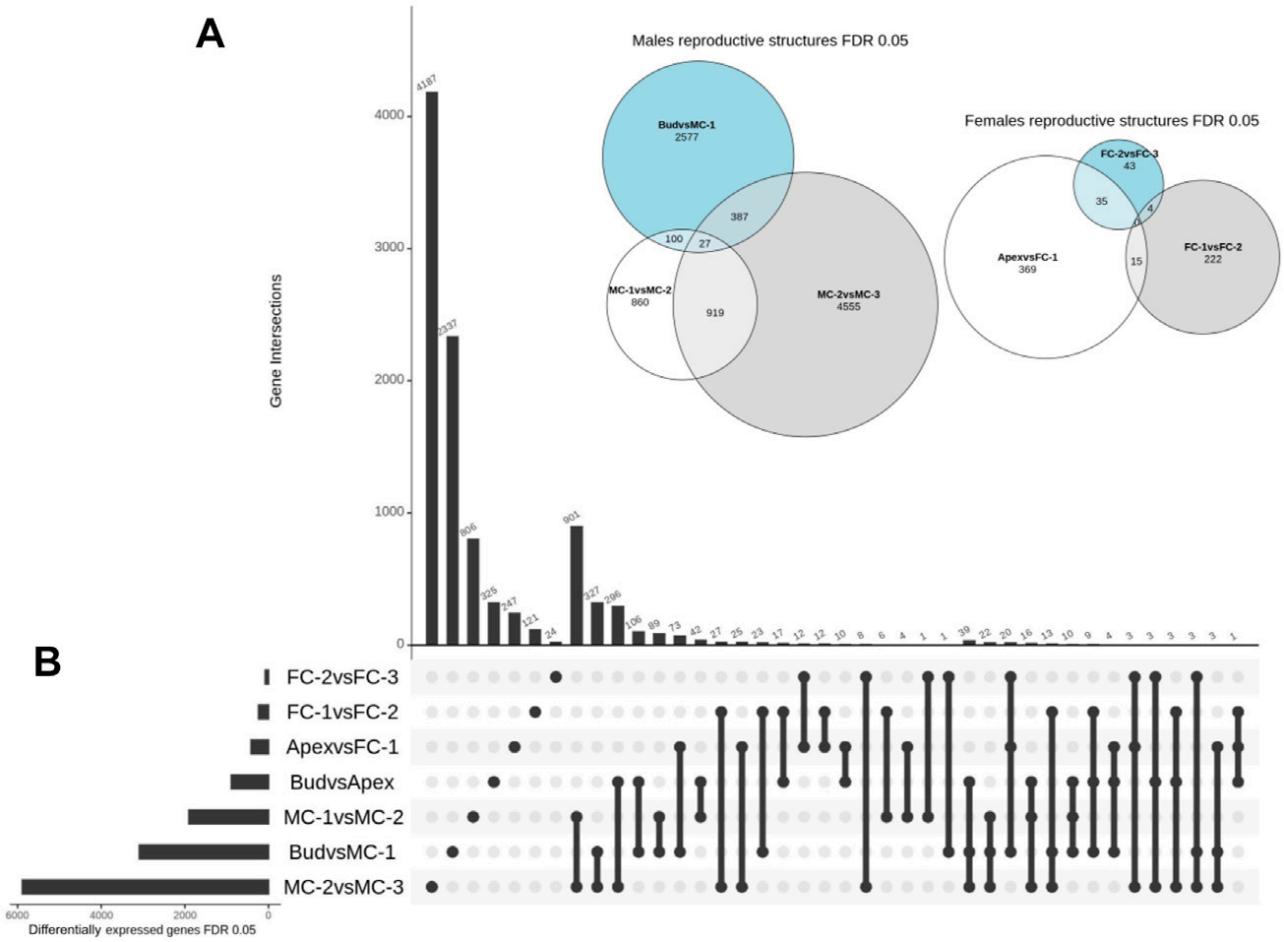
Overview of spatial and temporal differential gene expression. **(A)** Euler diagram showing the number and overlap of DEGs between the time points for each gender. Size of the bubble refers to the total number of DEGs and outer numbers represent DEGs only in this comparison. **(B)** UpSet plot summarizes the DEG overlap between all seven set comparisons. The horizontal bars show the number of differentially expressed genes identified by each comparison, while the vertical bars display the size of sets of genes identified by only one comparison and the intersection sets. Single dots and the corresponding horizontal bars represent the number of genes that are unique for that dataset and not shared between the other comparisons.

We then conducted a detailed comparative analysis of the DEGs using three combinations of stages that represented the female cone development (apex (AP) vs. FC-1, FC-1 vs. FC-2, FC-2 vs. FC-3) and three combinations representing male cone development (bud vs. MC-1, MC-1 vs. MC-2, MC-2 vs. MC-3). We found that the set including MC-2 vs. MC-3 had the highest number of unique transcripts (4,555) (Figure 3A). Lower numbers were identified in samples of the female cone time series, varying between 43 and 369 unique transcripts. The overall number of shared DEGs between the male cone developmental stages (MC-1 vs. MC-2/MC-2 vs. MC-3) was 919, 100 shared DEGs between the early stages (bud vs. MC1/MC-1 vs. MC-2) and 27 DEGs between all three combinations. The number of DEGs shared between female cones stages were 4, 15, and 35 in FC-1 vs. FC-2/FC-2 vs. FC-3, AP vs. FC-1/FC-1 vs. FC-2, AP vs. FC-1/FC-2 vs. FC-3, respectively, while no DEGs were found in all three combinations (Figure 3A). The number of shared transcripts between the two genders at an early time point (bud vs. MC-1 with AP vs. FC-1) was 73, whereas the number of transcripts in common in the groups ranged between one to 27 (Figure 3B).

**FIGURE 4:**
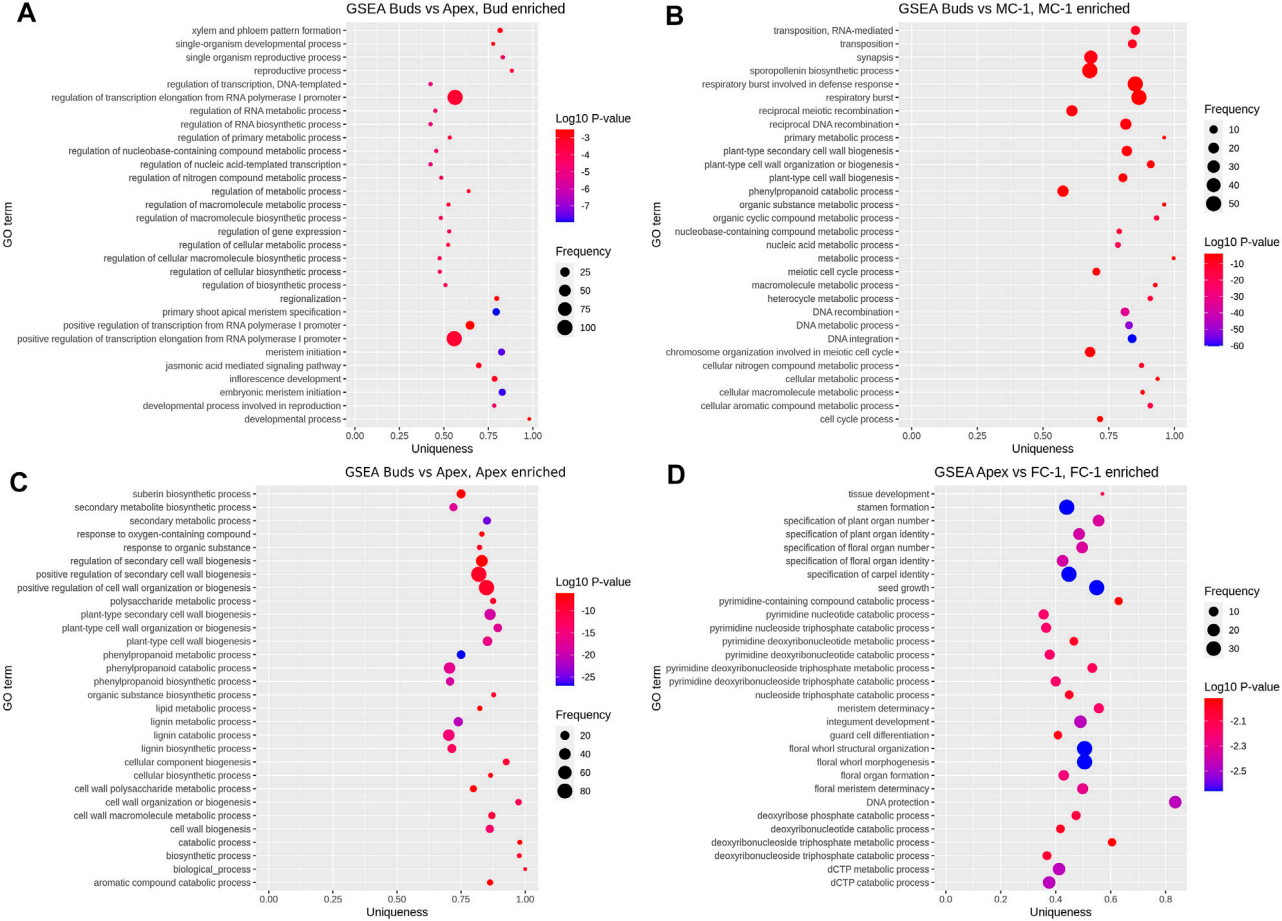
Gene set enrichment analysis. Showing top 30 GO terms enriched in each set of pairwise comparisons: **(A)** bud (enriched) vs. apex, **(B)** bud vs. MC-1 (enriched), **(C)** bud vs. apex (enriched), **(D)** apex vs. FC-1 (enriched). Vertical axis shows the GO terms and the horizontal axis represents the enrichment factor uniqueness which implies whether the term is an outlier when compared to the entire list. Colour change represents the Log10 *p*-value and size of the bubble represents the number of genes in the GO term (frequency) which was obtained by comparing the number of enriched terms to the uniprot database.

To further investigate the role of transcripts involved in the early development of reproductive structures we performed a gene set enrichment analysis based on the functional annotation of our DEGs. By comparing the frequency of GO annotations of the DEGs in FC-1 with the ones from apex (AP vs. FC-1) we found that a high number of enriched terms are especially related to floral identity and floral organ development in female cones (Figure 4D). In the top 30 enriched terms for the two early stages of male cones (bud vs. MC-1) we observed “sporopollenin biosynthetic process” to have a higher frequency (Figure 4B). However, we also compared the frequency of GO terms between apex and bud to investigate the difference between the transcriptomes of the tissue area where male and female cones appear. Here, the terms “meristem initiation,” “reproductive process,” “inflorescence development” and “developmental process involved in reproduction” were enriched in bud samples (Figure 4A). No flowering related GO annotations were found in the first 30 enriched terms in the apex samples (Figure 4C).

### 3.5 Functional Classification of Differentially Expressed Gene

**FIGURE 5:**
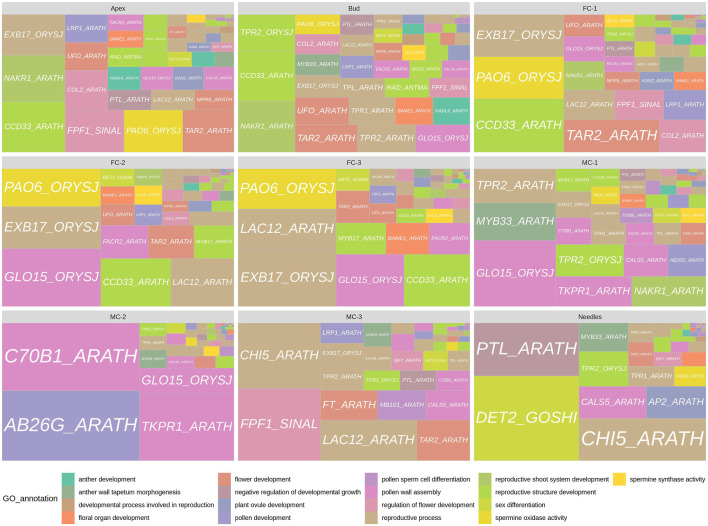
Panel of genes with floral related GO terms for each tissue. Gene labels are derived from PFAM transcripts annotation. GO annotations related to each gene label are represented by colors. The squares are differentially expressed transcripts annotated as the represented gene label and the size of the squares are equal to the sum of each transcript normalized counts over all replicates for the given sample.

To identify key regulators of early cone development, we combined the data of our GO enrichment analysis and the pairwise DEG comparisons. We selected DEGs with GO terms related to reproduction, regulation of flower initiation or development, and then analysed their expression profiles (Supplementary File S6). In six comparisons (bud vs. AP, bud vs. MC-1, AP vs. FC-1, AP vs. FC-3, FC-1 vs. FC-2, FC-1 vs. MC-1) we found 161 transcripts that met the profile of being both differentially expressed and having flowering related GO terms associated. In general, 68 of the 161 DEGs had more than one GO term assigned, many of which are ancestor or child terms to the corresponding GO. *P. densiflora* transcript *DAL14* (*MADS6_ORYSJ_1*) was associated with the most GO terms, 16 in total, and mostly related to floral organ formation. We found 62 DEGs associated with terms such as “reproductive process,” “regulation of flower development,” “developmental process involved in reproduction” or “reproductive shoot system development.” Forty one DEGs were assigned to general flower structure terms, while 29 DEGs had male structure related terms e.g. “pollen development” or “anther development,” etc., and five DEGs had female structure related GO terms, e.g. “plant ovule development” or “specification of carpel identity.” We found 63 DEGs with “response to gibberellin” or “response to abiotic stimulus” and two DEGs with the terms “negative regulation of developmental growth” and one with “sex differentiation” (Figure 5).

Subsequently, we investigated the temporal and spatial expression of transcripts, for instance DEGs that are up-regulated in individual stages or in similar tissue types during reproductive development. Transcripts with high expression in the early time points like buds, apex and FC-1 were found to be annotated to terms related to reproductive structure development (Figure 5). For example, we found that genes differentially expressed in buds were also differentially expressed in either apex/FC-1 or MC-1. Theseincluded a *WUSCHEL-RELATED-HOMEOBOX-3* (*WOX3_ARATH*), two *RAD-LIKE* genes (*RAD_ANTMA, RADL6_ARATH*) and three *TOPLESS-RELATED* genes (*TPR4_ARATH, TPR1_ARATH, TPL_ARATH*). Many of the genes co-expressed in apex, bud and FC-1 belonged to the MADS-box transcription factor family. Transcripts that are close relatives to *HEADING-DATE-3A*, a *KANADI-4* gene, and a *NUCLEAR-ENRICHED-PHLOEM-COMPANION-CELL-GENE-1* (*NAKR1_ARATH*) (Supplementary File S6), also were differential expressed at the early time points (apex/bud). In young female cones (FC-1) 16 out of 29 genes were associated to GO terms like “response to abiotic stimulus” and “response to gibberellin” (Supplementary File S6). Others were involved in either regulating “flower development” or “flower structure” such as a *CONSTANS-LIKE 2* gene or a *BLADE-ON-PETIOLE-2* gene. Many of the 29 genes were exclusively expressed in female cone related tissues and known to regulate flower formation or ovule development in angiosperms. We made similar observations with developmental samples FC-2 and FC-3. Most genes were involved in “flower structure,” “floral organ number” and “promotion of gynoecium,” “ovule” or “carpel specification.” These included MADS-box transcription factors like *MADS6_ORYSJ* (*DAL14*) or *MAD18_ORYSJ* (*DAL10*) and a *UNUSUAL-FLORAL-ORGANS* ortholog.

Genes that had a strong expression in male cones (MC-1-3) were associated with GO terms like “pollen development,” “sporopollenin biosynthesis,” “anther wall tapetum morphogenesis” or “pollen wall assembly.” However, four genes that were expressed the most in buds, MC-1 and/or MC-2 belong to the *TOPLESS-RELATED* (*TPR/TPL*) family. Family members are known to act as corepressors and modulate gene expression in many processes, including hormone signalling, stress responses, and the flowering time control (Causier et al., 2012). More surprising was the expression of a transcript in MC-3 similar to the *FLOWERING-LOCUS-T* (*FT*) gene, as well as, a transcript homologous to a *FLOWERING-PROMOTING-FACTOR-1* (*FPF1*), the latter also co-expressed in apex and FC-1. Until now unbeknownst in conifers, in Arabidopsis this small protein is involved in the gibberellin response in apical meristems during the transition to flowering (Kania et al., 1997; Melzer et al., 1999). Interestingly, differential expression of an ortholog of a steroid 5-alpha-reductase gene (*DET2_GOSHI*) in needles, was annotated to the GO term “sex differentiation” (Supplementary File S6). These plant steroidal hormones, termed brassinosteroids, play essential roles in plant vegetative and reproductive growth (Zheng et al., 2020).

### 3.6 Identification and Gene Expression Patterns of MADS-Box Transcription Factors

As we identified many genes belonging to the MADS box transcription factor family during the annotation process we wanted to further investigate their role during cone development. It is well established that MADS-box transcription factors play a pivotal role in regulating flower initiation and floral organ development in both angiosperms (De Bodt et al., 2003) and gymnosperms (Mouradov et al., 1999; Sundström et al., 1999; Winter et al., 1999; Carlsbecker, 2002).

In our dataset, we identified in total 95 sequences as MADS-box genes. From these, 54 sequences were grouped into the MIKC-C type family, containing the typical structural motifs, such as the K-box region and DNA binding domains (SRF). A subsequent phylogenetic analysis was then conducted to assign them to gene families and assess their relationship to MADS-box genes from *A. thaliana, Picea abies* and *P. tabuliformis*. The resulting tree grouped the MADS-box genes into eight clusters: *GpMADS4-like, AGL17, B-sister, DEF/GLO/GGM13-like, SVP/StMADS-11-like, AG, AGL6/SEP,* and *TMR3/SOC1/AGL14*. The added *LEAFY* and *NEEDLY* genes formed their own clade, as expected, and were used for rooting the tree. Four of the major clades of floral homeotic MADS-box genes that were identified in *P. densiflora* belong to the B, C/D, and E-class. The three clades *AGL17, TMR3/SOC1/AGL14* and *SVP/StMADS-11-like* are related to flowering promoter gene groups (Supplementary Figure S4). The biggest group in our dataset was the *TM3/SOC1/AGL14* family consisting of 18 representatives, while the *GpMADS4* family represented the smallest group with two genes only. We could classify several genes into the *AGL17* family but no annotated co-orthologs for these groups were available from *Picea abies* or *P. tabuliformis*. Overall, we found 21 *P. densiflora* genes that are co-orthologs to the conifer specific *DEFICIENS-AGAMOUS-LIKE* (*DAL*) genes of *Picea abies* or *P. tabuliformis*. Interestingly, we found six *P. densiflora* MADS genes (*GGM13_GNEGN_3, MAD23_ORYSJ_1, MAD23_ORYSJ_2, AGL9_PETHY, MADS6_ORYSJ_2, MADS6_ORYSJ_3*) that clearly separated from the conifer or Arabidopsis sequences. Subsequent protein sequence comparison with other sequences deposited in the NCBI database showed similarity only between 49.61 and 79.65% (Supplementary File S5).

**FIGURE 6:**
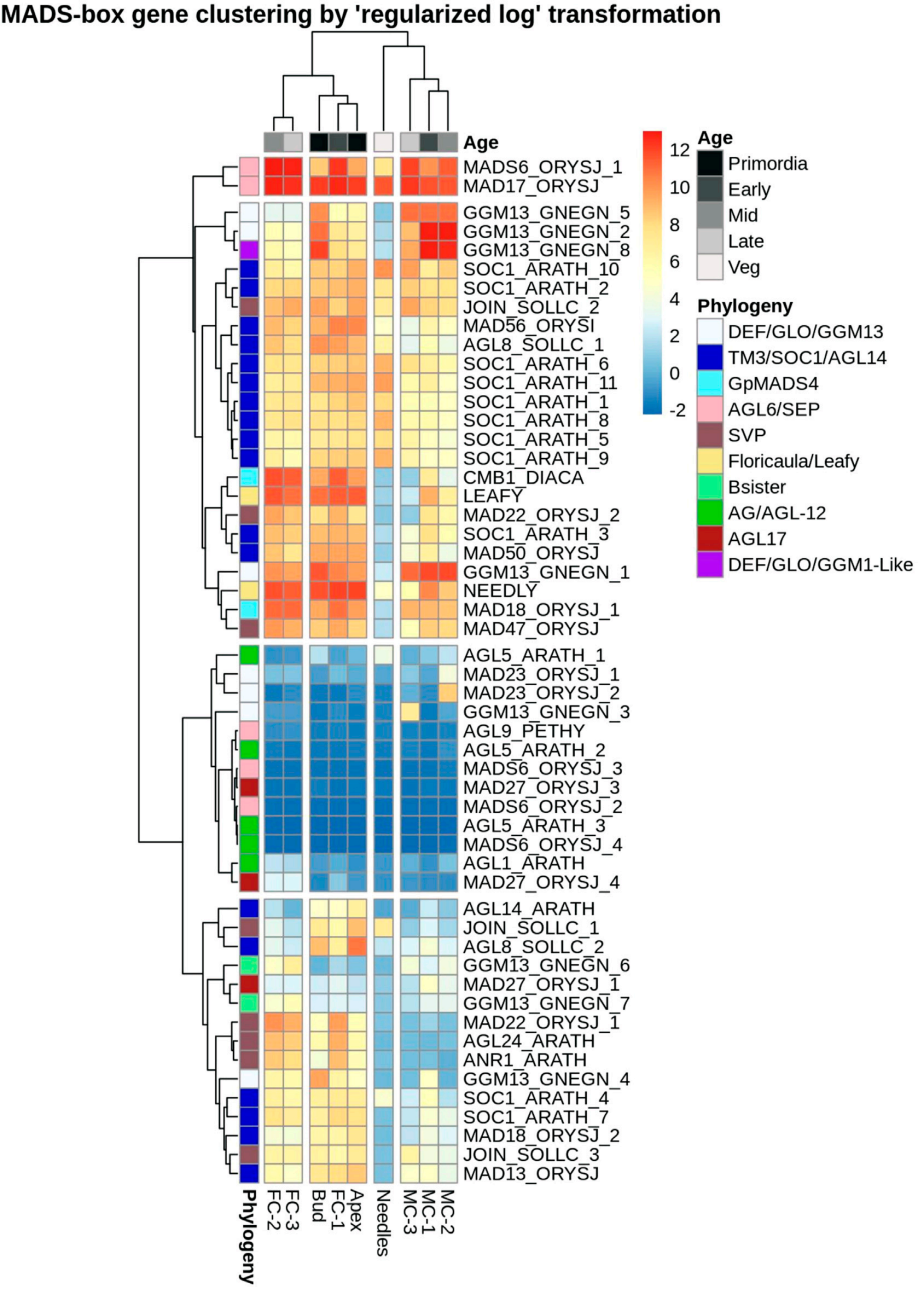
Heatmap showing expression levels of *P. densiflora* MADS-box type II genes hierarchically clustered based on relation to time-points and tissue types. Rows correspond to the 54 MADS box genes (names abbreviated to the closed angiosperm ortholog) and columns correspond to the nine tissue types. A relative decrease in expression (normalized counts) is indicated in blue while increases are red. Here, “Veg” is related to needles sampled regardless of stage. Genes with less than three counts in each independent dataset were omitted in this analysis.

Expression pattern analysis of these 54 MIKC-C family MADS-box genes revealed that they clustered into four main groups (Figure 6). One group was formed by *DAL14* (*MAD6-ORYSJ-1*) and *DAL1* (*MAD17-ORYSJ*) with high expression in all tissues. In a second group, genes were moderate to highly expressed in most of the tissue types. For example, orthologs of *DAL11* (*GGM13GNEGN5*), *DAL12* (*GGM13-GNEGN-1*) and *DAL13* (*GGM13-GNEGN-2/GGM13-GNEGN-8*) were up-regulated in male cones and buds compared to other tissues. *DAL21* (*CMB1-DIACA*), *DAL10* (*MAD18-ORYSJ*), *NEEDLY* and *LEAFY* were highly expressed in female cones, buds and apex than in needles and male cones. In the third group, seven MADS-box genes were low or not expressed in the analysed tissues. Genes homologous to *DAL20* (*AGL5-ARATH-3*), *DAL2* (*AGL9-PETHY*) and *DAL5* (*AGL5-ARATH-2*) were present in this group. We found five genes where expression was found only in a small number of tissue types and time points. For instance, homologs of *MAD23-ORYSJ-1* and *MAD23-ORYSJ-2* were only up-regulated in MC-2 while a *GGM13-GNEGN-3* homolog was only expressed in MC-3. Transcripts that were exclusively up-regulated in female cones in this third group were close relatives to *AGL1-ARATH* and *MAD27-ORYSJ-4* (Figure 6). In the last group, we found 15 MADS-box transcription factors that were up-regulated in female cone, bud and apex transcriptomes and only low to moderately expressed in other organs. These were transcripts such as *MADS22-ARATH-1* or *DAL12* (*GGM13-GNEGN-4*). Specifically, up-regulated in apex, bud and FC-1 samples was a set of four genes (*AGL14-ARATH, JOIN-SOLLC-1, AGL8-SOLLC-2, MAD18-ORYSJ-2*), all members of the flowering promoters *StMADS-11-like* or *TM3/AGL14* group. In summary, most of the MIKC-C MADS box genes identified in *P. densiflora* showed general but also individual expression patterns specific to male and female cone samples and time points.

## 4 Discussion

The transition from vegetative to reproductive growth and the formation of an inflorescence is a distinctive morphological process that has been well characterised in many angiosperm species such as Arabidopsis and poplar. In contrast, forecasting which of the many vegetative meristems will transition to a floral meristem in gymnosperms is a difficult task, since this process is environment dependent, species-specific and occurs irregular even within the same tree species, but, is under the control of a set of genes and transcription factors (Mouradov et al., 1998). To partially overcome this challenge, we selected the highly floriferous conifer, *P. densiflora* for our transcriptome study, and provide transcriptomes of several male and early female cone development stages. We then focused on the MADS-box type II genes and DEGs linked to flowering related GO annotations to identify new regulators involved in conifer cone development.

The combined genome guided and *de novo* assembly approach used in our study resulted in a low percentage of fully annotated sequences. Typically, an angiosperm genome assembly is expected to have a classification rate of 40–50% and annotation rate of 70% (Bolger et al., 2018; Schwacke et al., 2019), whereas we report values of 19 and 32%, respectively. These findings are in line with the hypothesis that multiple partial transcript assemblies remain as representative sequences. When compared with other publicly available conifer transcriptome-derived protein sets, our data competed well or outperformed other transcriptome assemblies (Zhang et al., 2012; Uddenberg et al., 2013; Canales et al., 2014; Elbl et al., 2015; Niu et al., 2015; Little et al., 2016; Duan et al., 2017; Mo et al., 2019). Furthermore, comparative analysis using Mercator showed that whilst there was at least one transcript for 4,182 classes for the *P. densiflora* assembly, the number of one transcript per class was lower at 3,307, 4,003, and 3,439 for *P. taeda, P. pinaster*, and *Picea abies*, respectively (Supplementary File S2). Taken all together these findings suggest our work has produced a high-quality transcriptome. Cluster-dendrogram analysis (Figure 2A) showed that the transcript profiles of most sample replicates are highly correlated to each other. However, one MC-1-replicate formed an outlier, clustering closer to the bud samples rather than the other MC-1 replicates. Analysis of samples by microscopy suggested that a few samples collected as buds may have already initiated reproductive development (Supplementary Figure S5). As we sampled three non-clonal trees it is not surprising that some samples had differences in their developmental stage. In general, the libraries from our samples separated spatially and temporally, confirming that our sample types and time points had enough differences in their transcription profiles to cluster into independent groups. Less differences in transcript expression occurred between the stages of early female cone development (apex/FC-1), later female stages (FC-2/FC-3) and the male cone stages (MC-1/MC-2), respectively, as these libraries clustered close to each other.

Identification of DEGs with GO term annotations associated to floral functions provide a route to identify genes associated with cone development. DEGs in early female cone stages included transcripts orthologous to *PETAL-LOSS* (*PTL_ARATH*), *KANADI-4* (*KAN2_ARATH*), *KANADI-2* (*KAN2_ARATH*), *RAD-LIKE* gene (*RAD_ANTMA*) or *UNUSUAL-FLORAL-ORGANS* (*UFO_ARATH*). All of which we postulate are involved in female organ development, floral patterning or symmetry due to their expression and GO term classification. In male cone samples, we identified several genes associated with GO terms like pollen wall assembly (e.g., *TKPR1_ARATH*) or pollen cell (e.g., *KN1_ARATH*), as well as belonging to the conserved *TOPLESS-RELATED* (*TPL/TPR*) family. In angiosperms *TPL/TPRs* are central repressors interacting with partners from circadian rhythm, hormone signalling and other developmental pathways (Osmont and Hardtke, 2008; Wang et al., 2013; Martin-Arevalillo et al., 2017). It has been shown that TPR/TPL proteins are not only able to bind specific regions of the *CO* and *FT* promoters, thus manipulating flowering time, but are also part of a repressor protein complex that is involved in the control of organ size in Arabidopsis (Goralogia et al., 2017; Li et al., 2018), making them interesting candidates for further functional studies.

Of particular interest were two DEGs, one annotated as *NUCLEAR-ENRICHED-PHLOEM-COMPANION-CELL-GENE-1* (*NAKR1_ARATH*) and a second gene annotated as *FLOWERING-PROMOTING FACTOR-1* (*FPF1_SINAL*). The first gene, *NAKR1_ARATH*, has been shown in angiosperms, through co-expression and direct physical interaction with the FT protein (Negishi et al., 2018; Zhu et al., 2016), to function as a phloem transporter that is required for the delivery of *FT* to the shoot apice However, no *FT* but only *FTL* genes have been reported in gymnosperms so far making the presence of the NAKR1 transporter gene in our sample set an interesting discovery. In this study we found two genes annotated as *HEADING-DATE-3A* (*HD3A_ORYSJ*) an ortholog of the angiosperm flowering promoting FT gene. One of the *HD3A_ORYSJ* genes appeared to be co-expressed at the same early timepoint as *NAKR1_ARATH*. Co-expression of spruce orthologs *NAKR1* (MA_525649g0010) and *PaFTL1* can also be seen on the public Diurnal Tools platform (https://diurnal.sbs.ntu.edu.sg/). The second gene of interest is a putative homologue of Arabidopsis, *FPF1_SINAL*. In Arabidopsis it encodes for a small protein that is able to shorten the vegetative phase and to induce precocious flowering when overexpressed by interacting with floral meristem identity genes and the gibberellin signalling pathway (Kania et al., 1997; Melzer et al., 1999). It has been demonstrated that *FPF1* acts downstream of *FT* in the long-day flowering response pathway and is also indirectly regulated by vernalisation (Greenup et al., 2010). Overexpression led to changes in wood development but not to early flowering in the perennial tree species poplar (Hoenicka et al., 2012).To our knowledge neither, *NAKR1* or *FPF1*, have been reported in conifers. Their GO term annotation as well as their differential expression between tissue types shown in this study indicates that both genes might be important for floral meristem fate and reproductive structure development in *P. densiflora*.

The number of *P. densiflora* type II MIKC-C MADS-box transcription factors identified in the current study (54 genes) was similar to that reported for other gymnosperms, including *P. taeda* (59 genes) and *Picea glauca* (58 genes) (Sundström et al., 1999; Shindo et al., 1999; Mouradov et al., 1999, 1998; Rutledge et al., 1998; Tandre et al., 1998, 1995; Becker et al., 2000; Nystedt et al., 2013; Gramzow et al., 2014; Zhao et al., 2015; Niu et al., 2016; Chen et al., 2019). Based on the phylogenetic analysis of these 54 MADS-box genes we defined eight clusters that were categorised into the B, C, D, and E-like gene and flowering promoter clades (SVP, AGL17, GpMADS4, AGL14/TM3/SOC1), confirming previous reports of the presence of these gene families in gymnosperms (Chen et al., 2019; Gramzow et al., 2014) (Supplementary Figure S4). Six *P. densiflora* MADS-box genes did not cluster with any of the sequences from closely related conifer species or Arabidopsis but within a gene family (Supplementary Figure S4). Three of the six genes (*GGM13_GNEGN_3, MAD23_ORYSJ_1, MAD23_ORYSJ_2*) clustered within the SVP subgroup, but had a low percent identity with other sequences deposited in the NCBI database (Supplementary File S5). These genes were each expressed in a single male cone time point (Figure 6) which is comparable with evidence found in angiosperms where B-class members are primarily expressed in microsporophylls and are involved in determining male gender as well as petal and stamen specifications (Angenent and Colombo, 1996; Weigel and Meyerowitz, 1994; Zahn et al., 2005). The other three transcripts (*AGL9_PETHY, MADS6_ORYSJ_2, MADS6_ORYSJ_3*) clustered with the MADS-box E-class group, that have floral organ identity determination functions in *Arabidopsis* (Supplementary Figure S4). Other MADS-box genes identified in our study were expressed in several tissues, both male and female structures as well as in the buds and apex samples. Few transcripts were solely expressed in samples collected at an early developmental time point (bud, apex, FC-1 and MC-1); *AGL8_SOLLC_2, MADS18_ORYSJ_2 and JOIN_SOLLC_1*. These genes are orthologs of Arabidopsis *AGL20* and *AGL24* where they have flower signalling functions. Their early time point expression in *P. densiflora* samples indicates that they might share similar functions. The first MADS box genes implicated in gymnosperm floral development were three DEFICIENS-AGAMOUS-LIKE (DAL) genes *DAL1, DAL2, DAL3*) from Norway spruce (Tandre et al., 1995). Most of the *P. densiflora DAL* genes identified in this study displayed similar expression patterns to the corresponding *DAL* genes described previously in gymnosperms (Chanderbali et al., 2010; Melzer et al., 2010; Chen et al., 2017; De La Torre et al., 2020). This suggests conserved roles of orthologs within the Pinaceae family (Tandre et al., 1998; Mouradov et al., 1999; Shindo et al., 1999; Winter et al., 1999; Sundström and Engström, 2002; Becker and Theißen, 2003; Carlsbecker et al., 2013; Liu et al., 2015; Niu et al., 2016). For example, *DAL11* and *DAL13* were highly expressed in male cones and bud samples, and have been previously associated with the specification of pollen cone identity (Carlsbecker et al., 2013; Niu et al., 2016). Also, several transcripts highly expressed in female structures including *DAL21, DAL22* and *DAL23* had been previously implied as key players in seed cone identity and development (Carlsbecker, 2002; Carlsbecker et al., 2003, 2013). An exception were the *DAL12* co-orthologs identified in this study that were expressed in all tissues, rather than exclusively in male tissues as reported in *Picea abies* (Carlsbecker, 2002; Sundström and Engström, 2002) indicating functions beyond male cone development in pines.

## 5 Conclusion

This work used the highly floriferous species *P. densiflora* to investigate the transcriptional changes during cone development without using chemical stimuli or mutant phenotypes. The distinct transcriptomes of the developmental stages that we produced from both female and male cones allowed us to identify DEGs related to reproduction. These genes may play roles in regulating flower initiation or structure development. Further analysis allowed us to identify which of the large number of MADS-box and conifer specific *DAL* transcription factors were associated with particular developmental processes. Taken together, our work provides a comprehensive genetic resource for conifer reproductive structure development which will be important for further molecular research and the biotech-based development of conifers with modified reproductive development.

## Data Availability

The datasets generated for this study can be found in the NCBI SRA database, BioProject ID: 497 PRJNA774359, https://www.ncbi.nlm.nih.gov/sra/PRJNA774359.
