# Supplementary figures and images for: Transcriptional Regulation of Pine Male and Female Cone Initiation and Development: Key Players Identified Through Comparative Transcriptomics
Source: Front Genet. 2022 Mar 18;13:815093. doi: 10.3389/fgene.2022.815093 (PMC8971679; doi:10.3389/fgene.2022.815093)

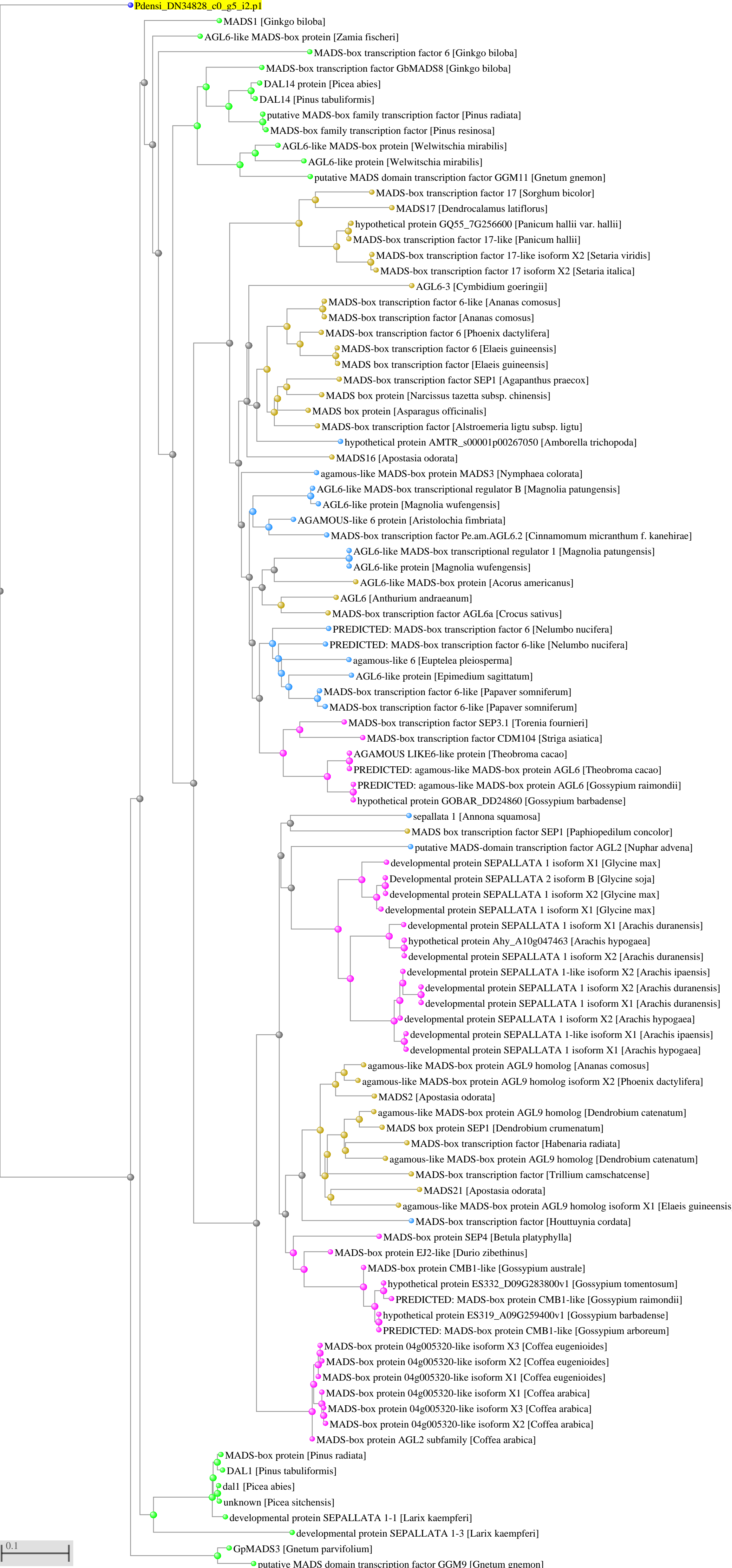

0.1

Supplement: Supplementary file 4 [file DataSheet4.ZIP › AGL9_PETHY.pdf]

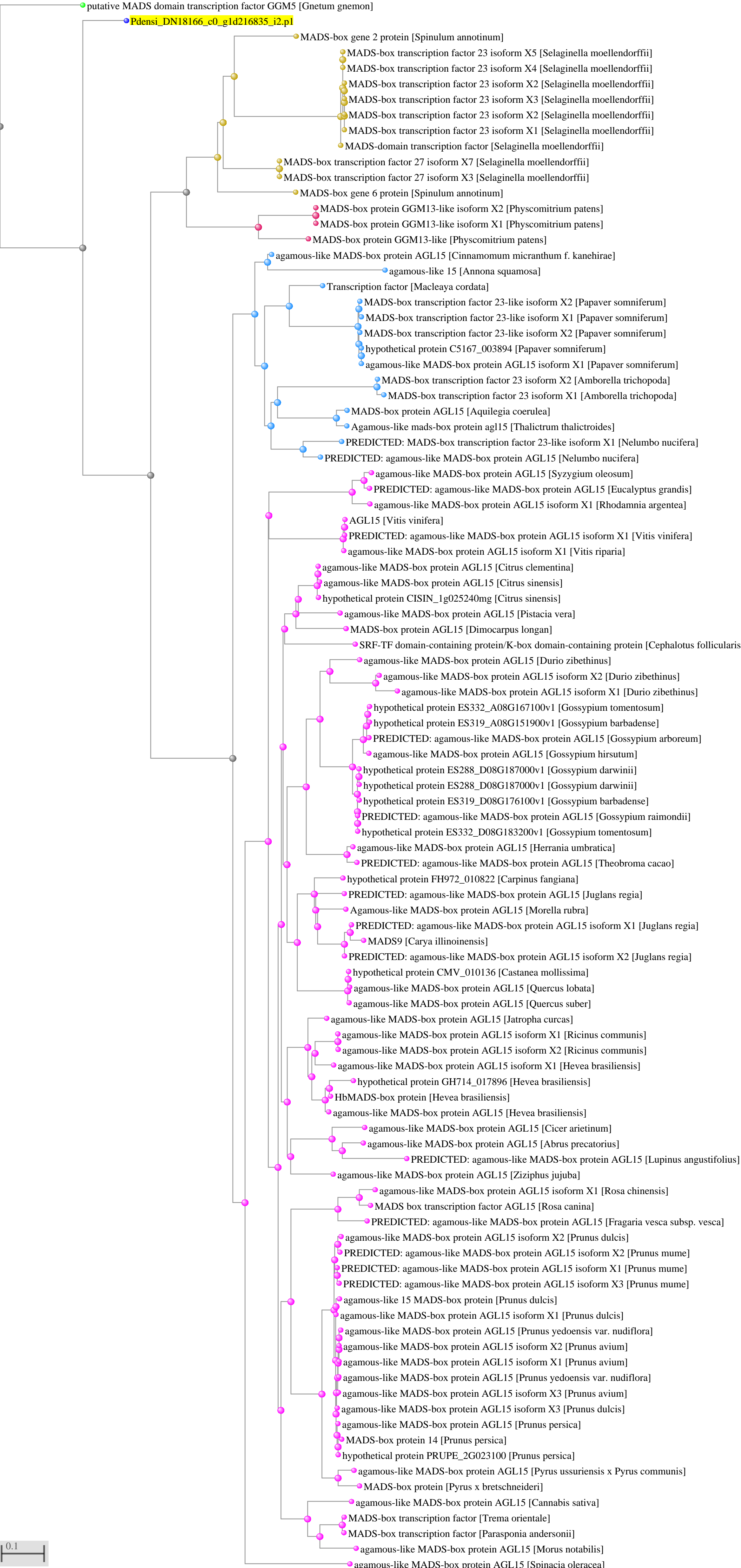

0.1

Supplement: Supplementary file 4 [file DataSheet4.ZIP › GGM13_GNEGN_2_aln.pdf]

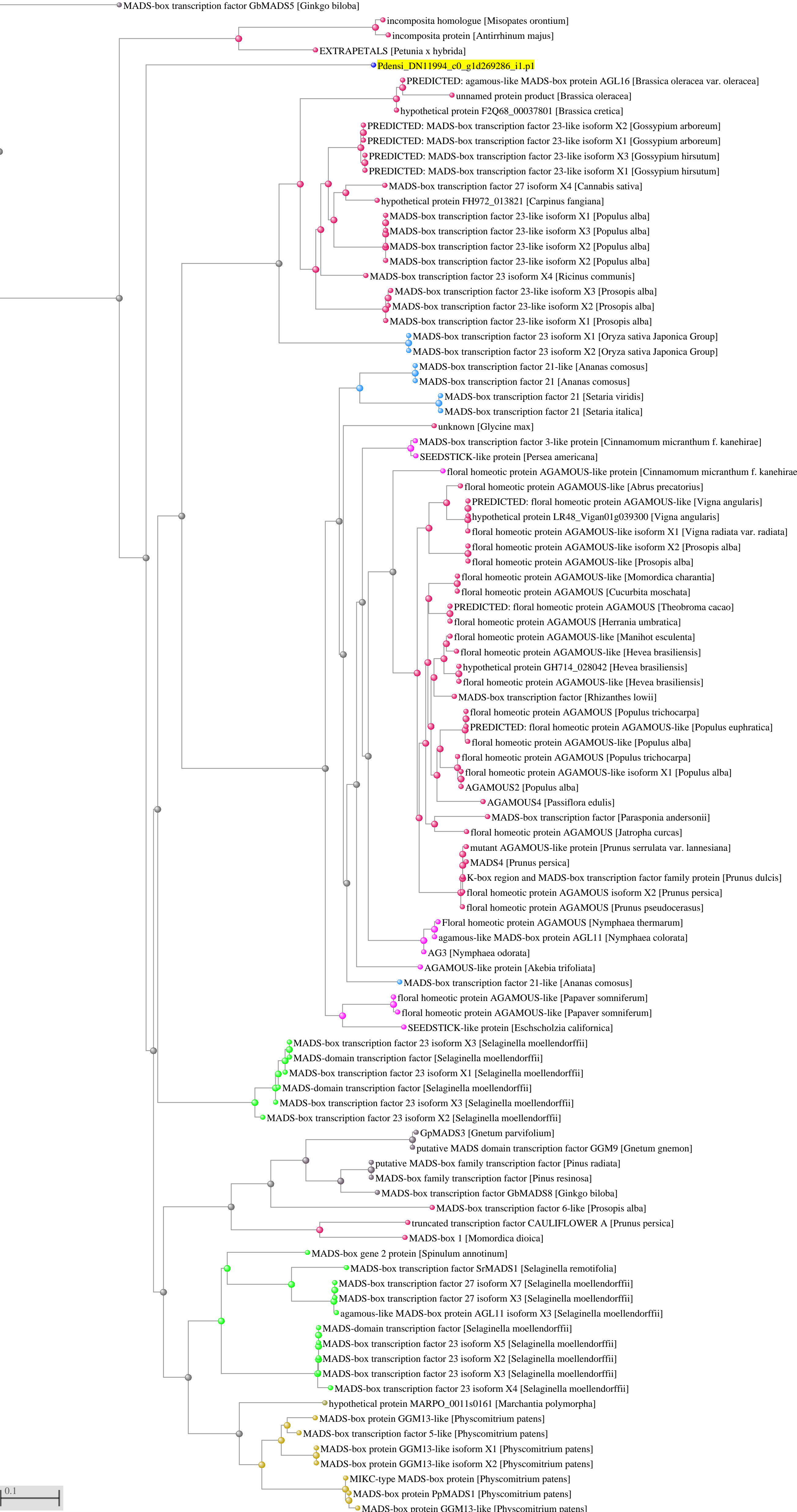

0.1

Supplement: Supplementary file 4 [file DataSheet4.ZIP › MAD23_ORYSJ_1.pdf]

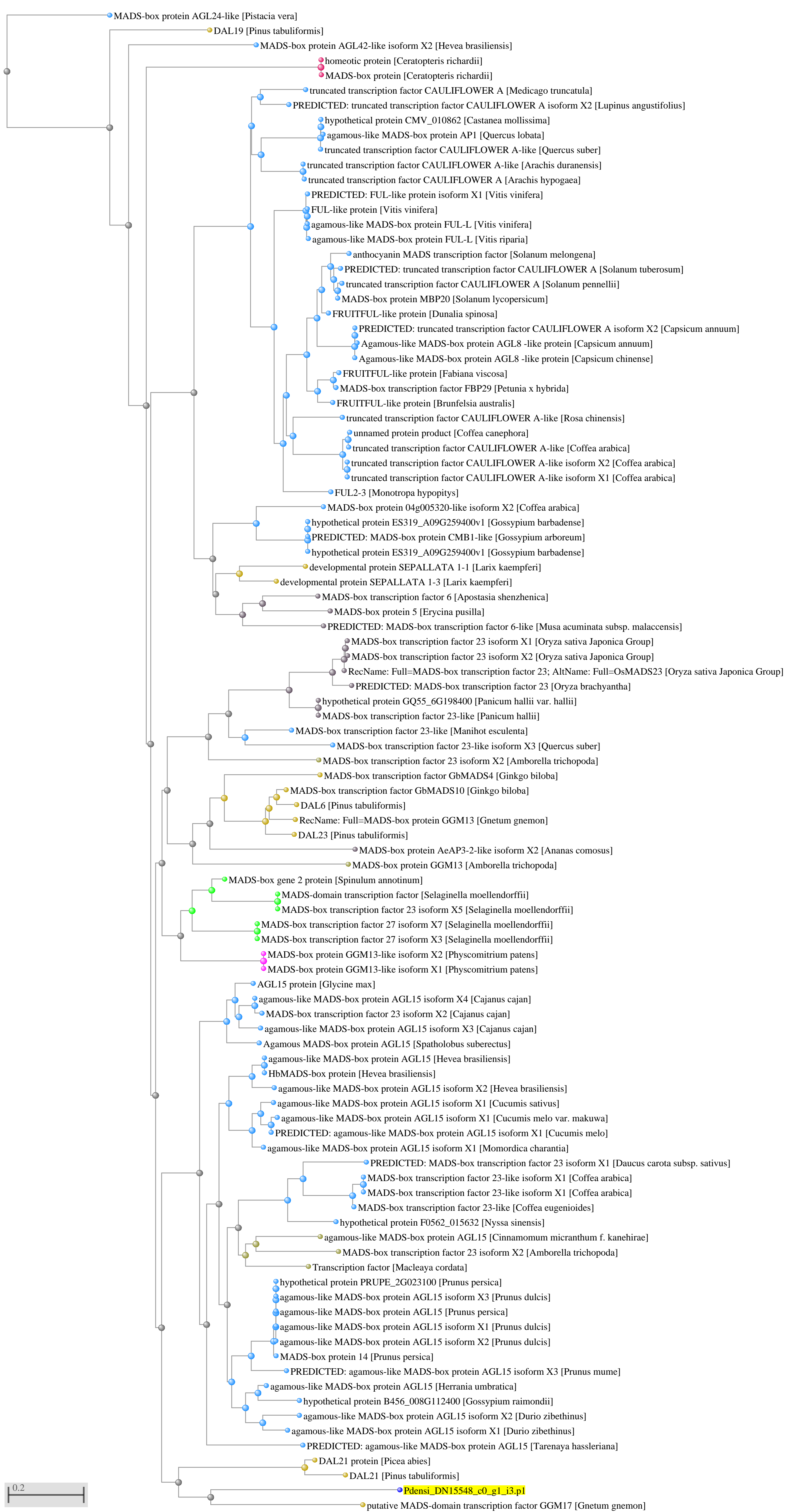

Supplement: Supplementary file 4 [file DataSheet4.ZIP › MAD23_ORYSJ_2.pdf]

Pdensi\_DN34828\_c0\_g2\_i3.pl

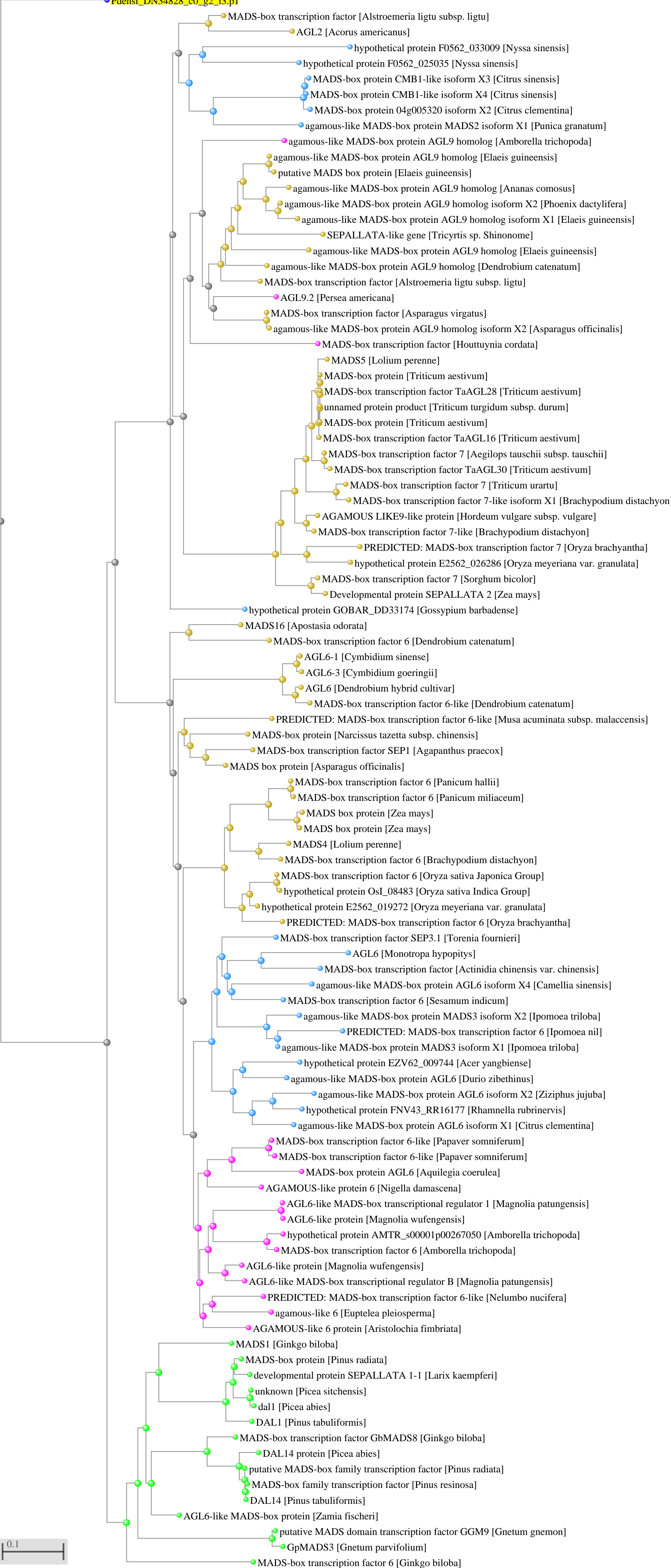

0.1

Supplement: Supplementary file 4 [file DataSheet4.ZIP › MADS6_ORYSJ_2.pdf]

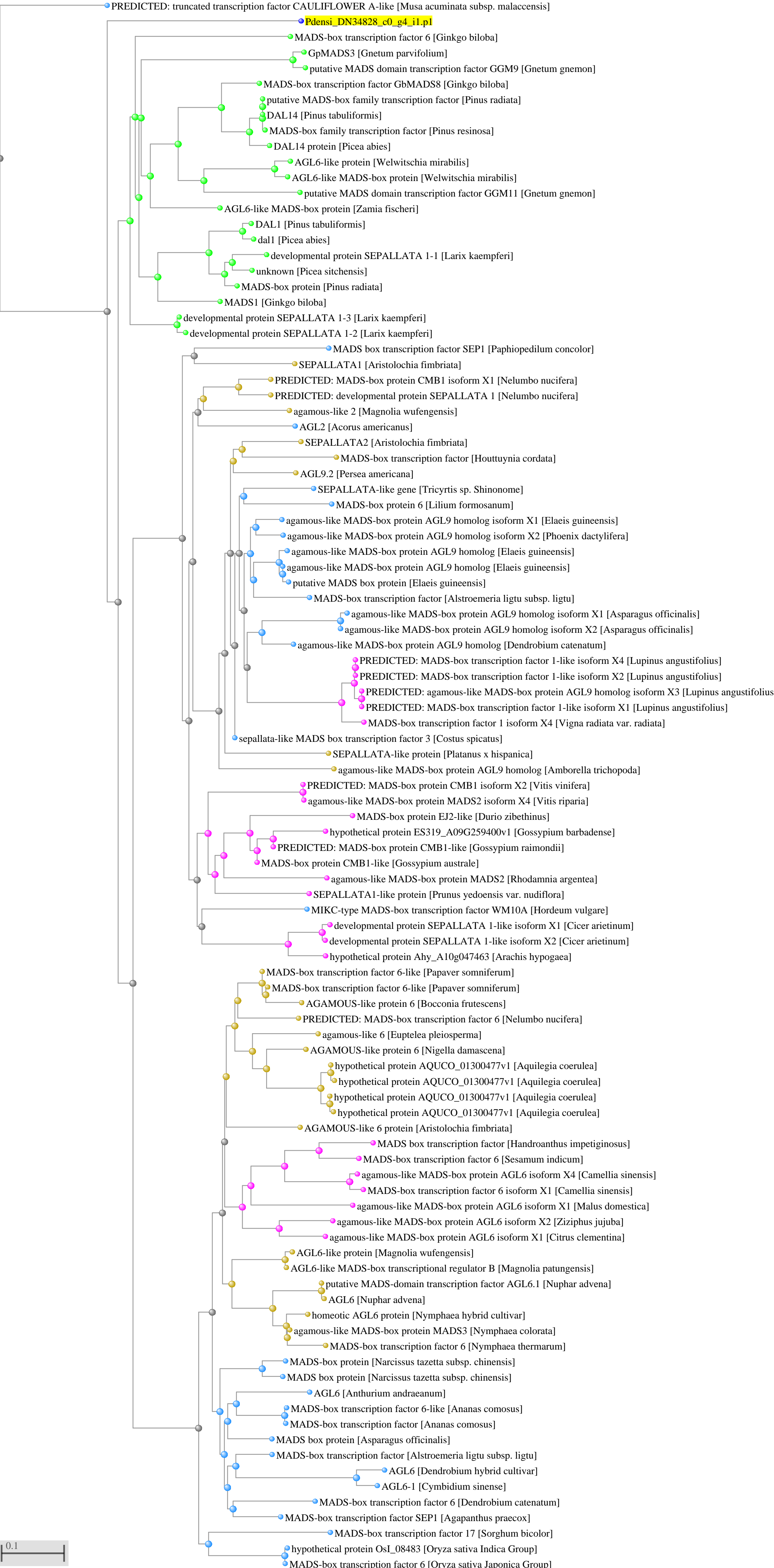

0.1

Supplement: Supplementary file 4 [file DataSheet4.ZIP › MADS6_ORYSJ_3.pdf]

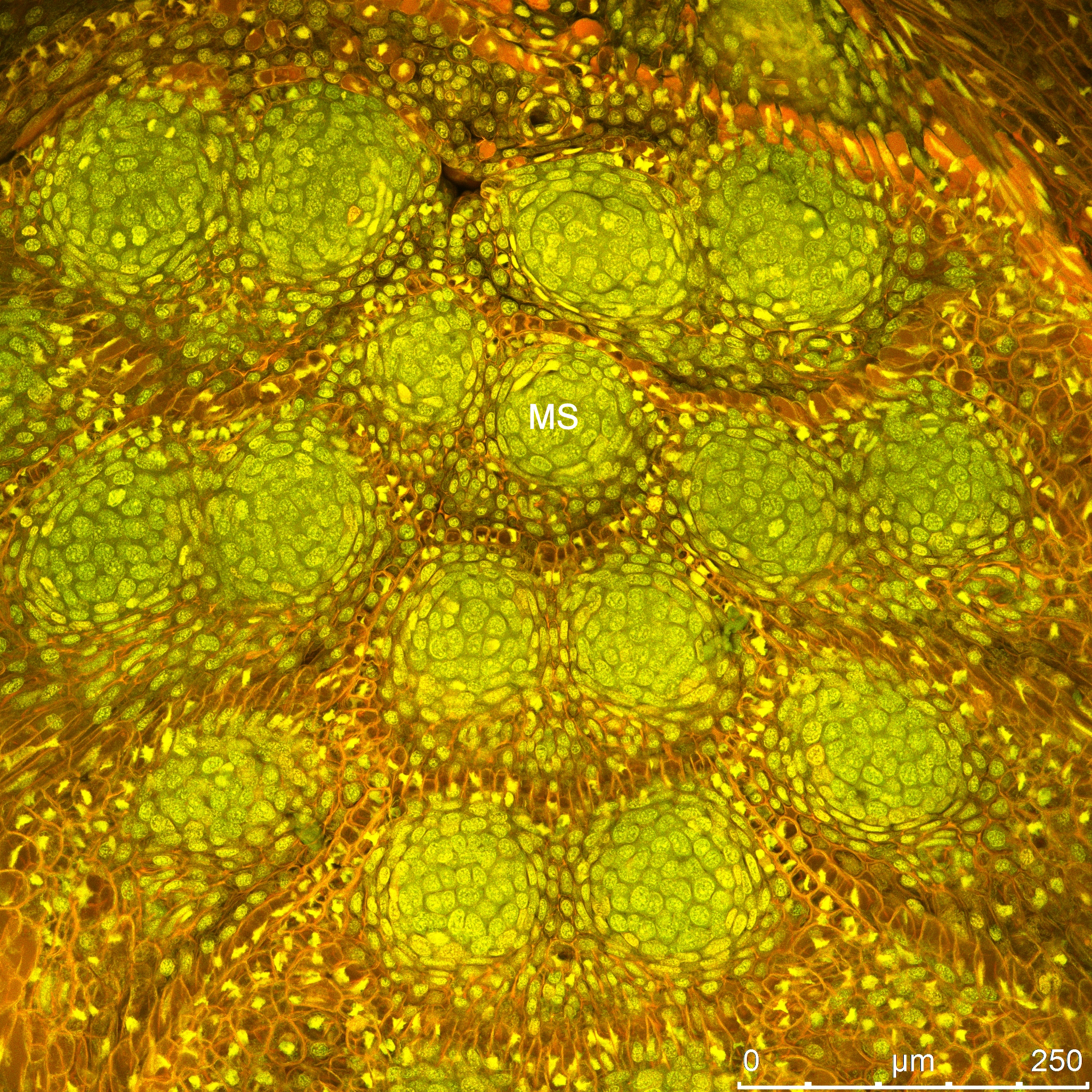

Supplement: Supplementary file 5 [file Image5.JPEG]

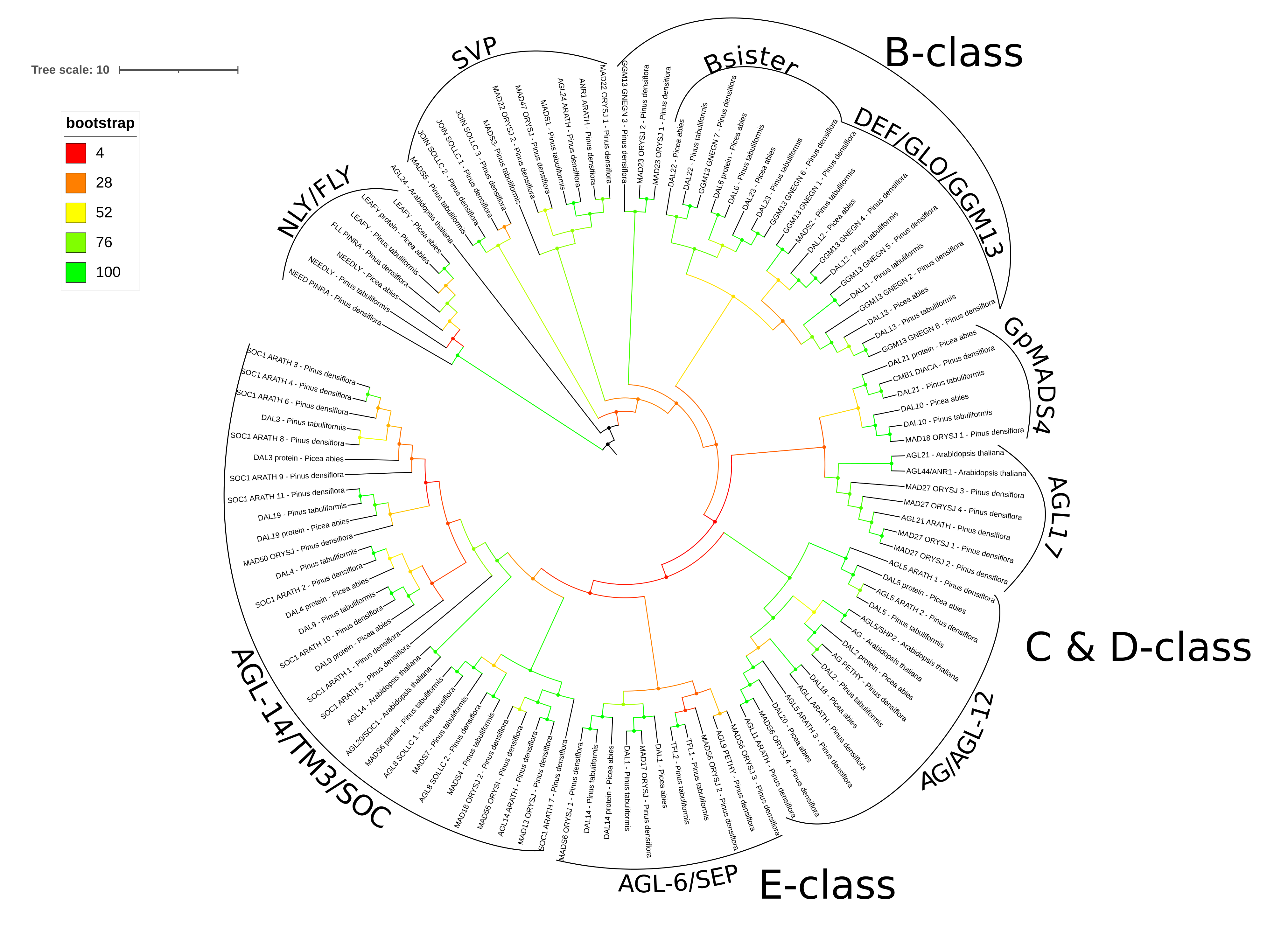

Supplement: Supplementary file 6 [file Image4.PNG]

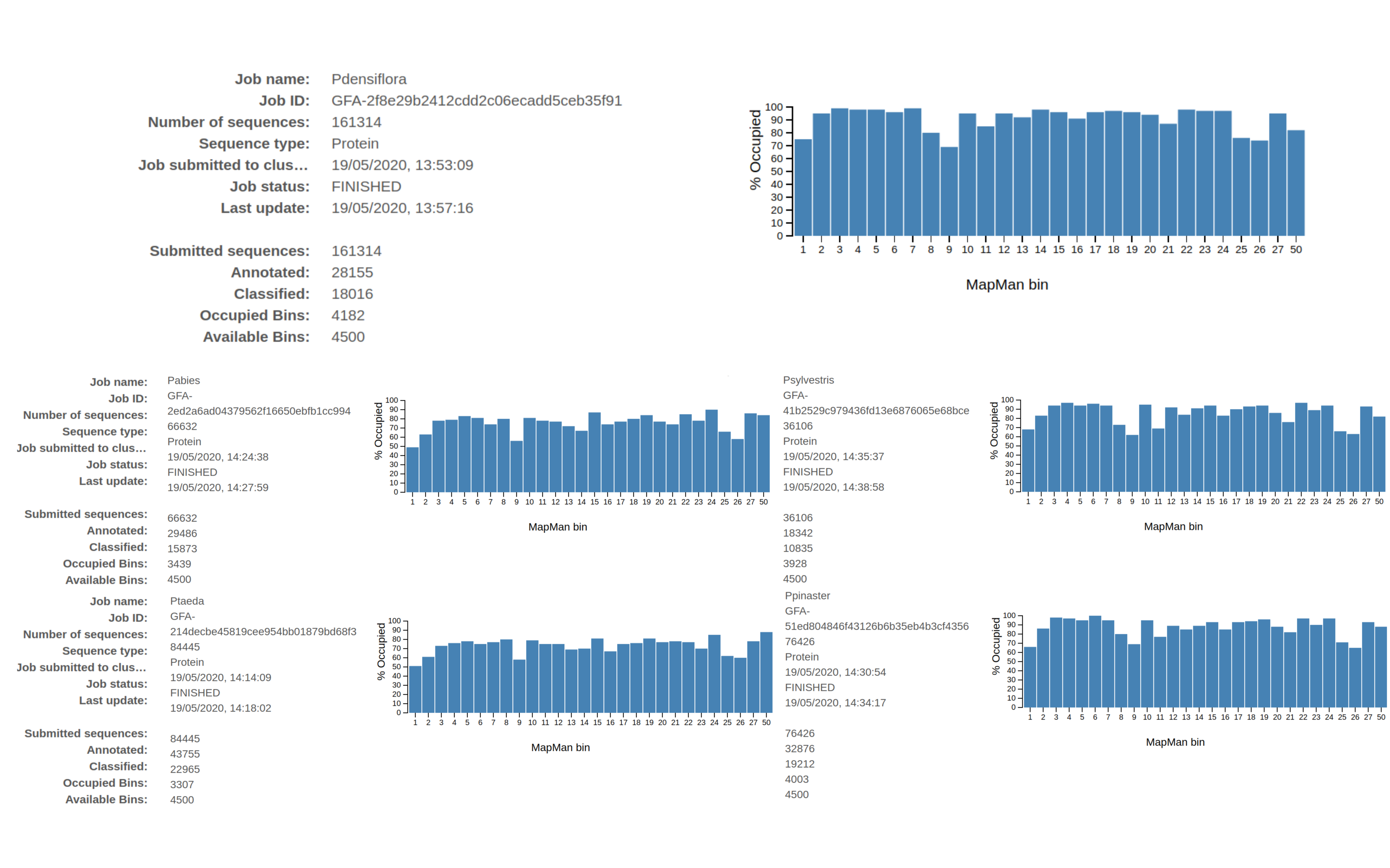

Supplement: Supplementary file 10 [file Image2.PNG]

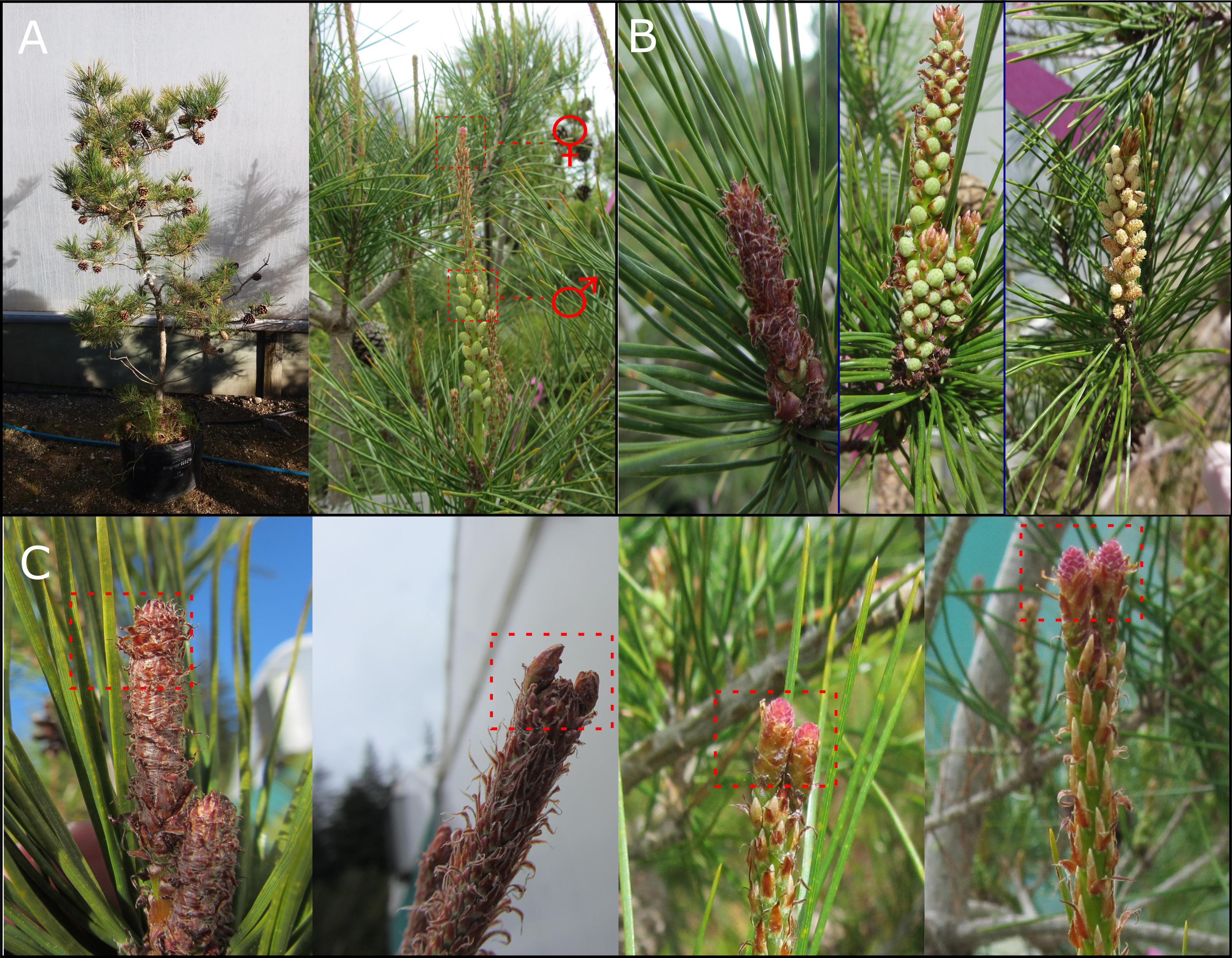

Supplement: Supplementary file 12 [file Image1.PNG]

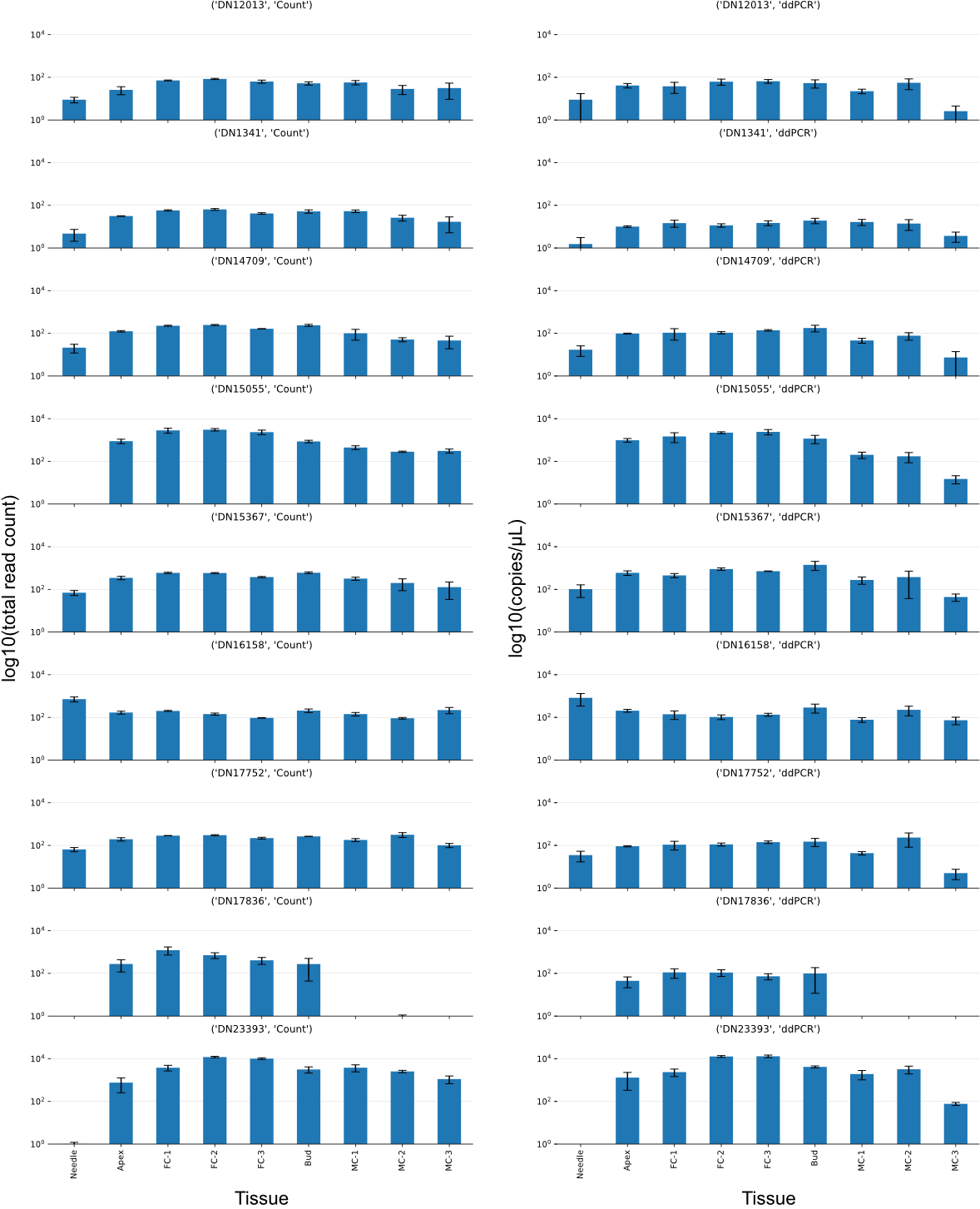

Supplement: Supplementary file 13 [file Image3.PNG]
