# Supplementary material for: Transcriptional Regulation of Pine Male and Female Cone Initiation and Development: Key Players Identified Through Comparative Transcriptomics
Source: Front Genet. 2022 Mar 18;13:815093. doi: 10.3389/fgene.2022.815093 (PMC8971679; doi:10.3389/fgene.2022.815093)
Supplement: Supplementary file 7 [file DataSheet1.PDF]

frontiers

# *Supplementary Material*

## 1 SUPPLEMENTARY DATA

### 1.1 Additional file: Supplementary file 1.

Gene names and oligonucleotides used in ddPCR.

### 1.2 Additional file: Supplementary file 2.

Mercator annotation comparison table. Output of Mercator annotation tool comparing the proteomics resource of *P. taeda, P. pinaster, Picea abies,* and *P. sylvestris*.

### 1.3 Additional file: Supplementary file 3.

Annotation file for all assembled transcripts.

### 1.4 Additional file: Supplementary file 4.

This files includes a compressed folder with pairwise DEG and GSEA analysis.

### 1.5 Additional file: Supplementary file 5.

New MADS. Blast results and phylogenetic trees for the six new MADS-box genes found in our dataset.

### 1.6 Additional file: Supplementary file 6.

All DEGs with GO terms related to reproduction, regulation of flower initiation or development, and their expression profiles..

### 1.7 Additional file: Supplementary file 7.

Sequencing read datasets statistic.

## 2 SUPPLEMENTARY FIGURES

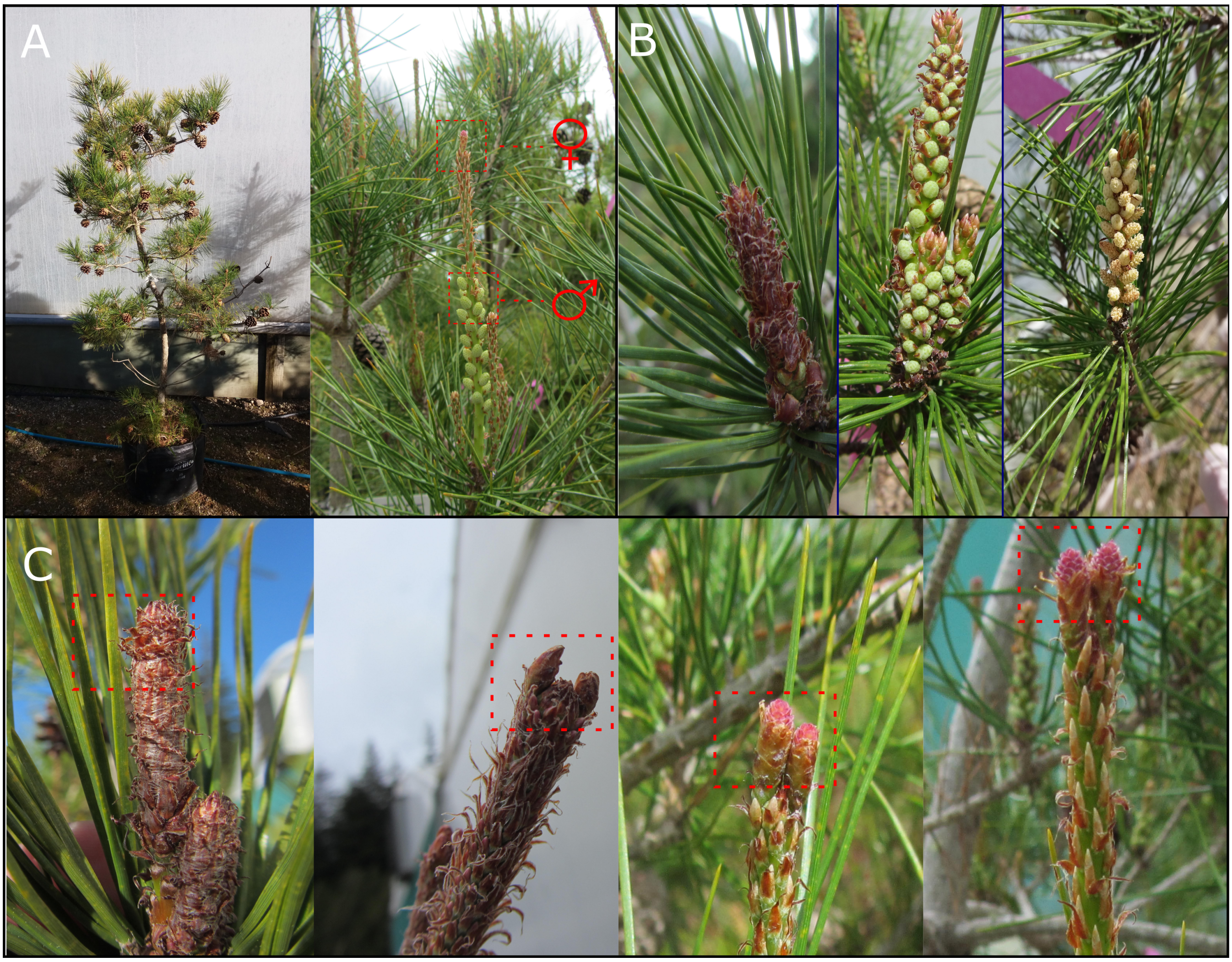


**Figure S1.** Sampling for *Pinus densiflora* reproductive structures. **(A)** Whole tree and location of male and female cones on a lateral shoot. **(B)** From left to right showing male cone samples *in situ* corresponding to the sampling types: MC-1, MC-2, MC-3. **(C)** From left to right showing in the red boxes female cone samples: apex, FC-1, FC-2, FC-3.

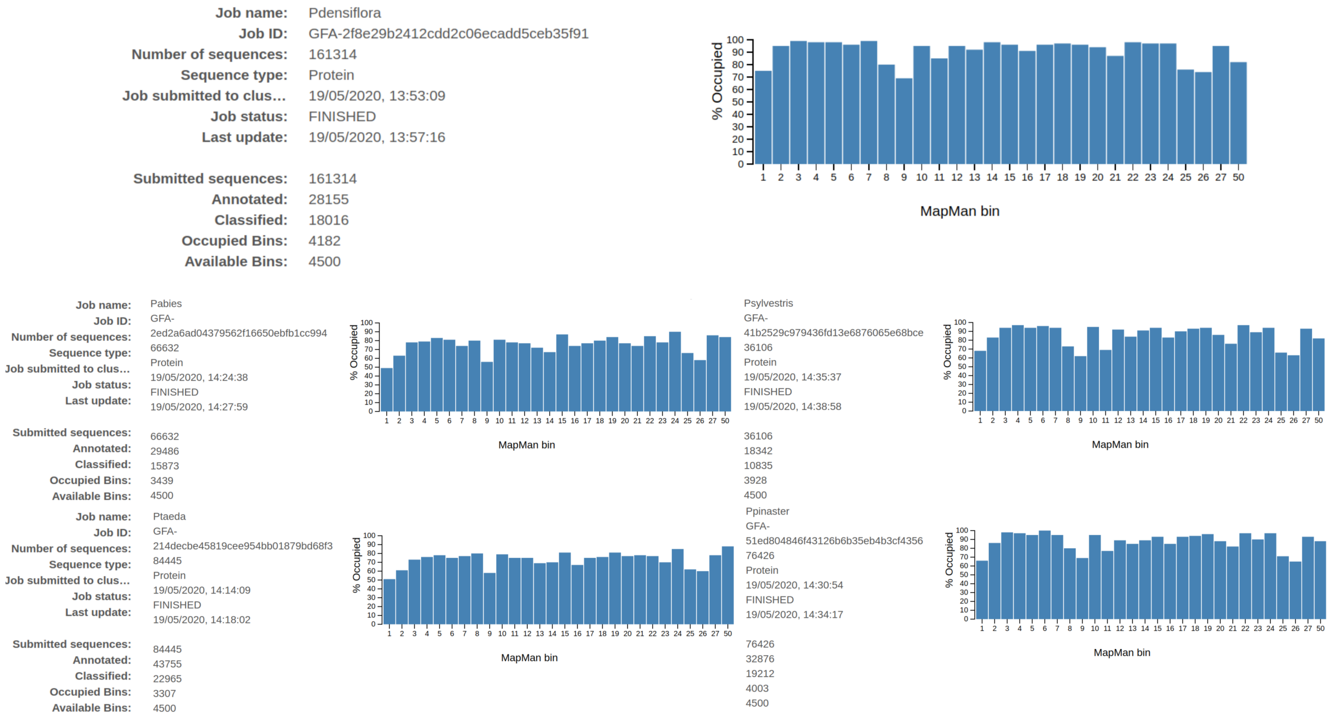


**Figure S2.** Mercator annotation tool. The occupancy rate of *Pinus densiflora* is higher when compared to public available proteomes, evidenced here by the occupied bin numbers.

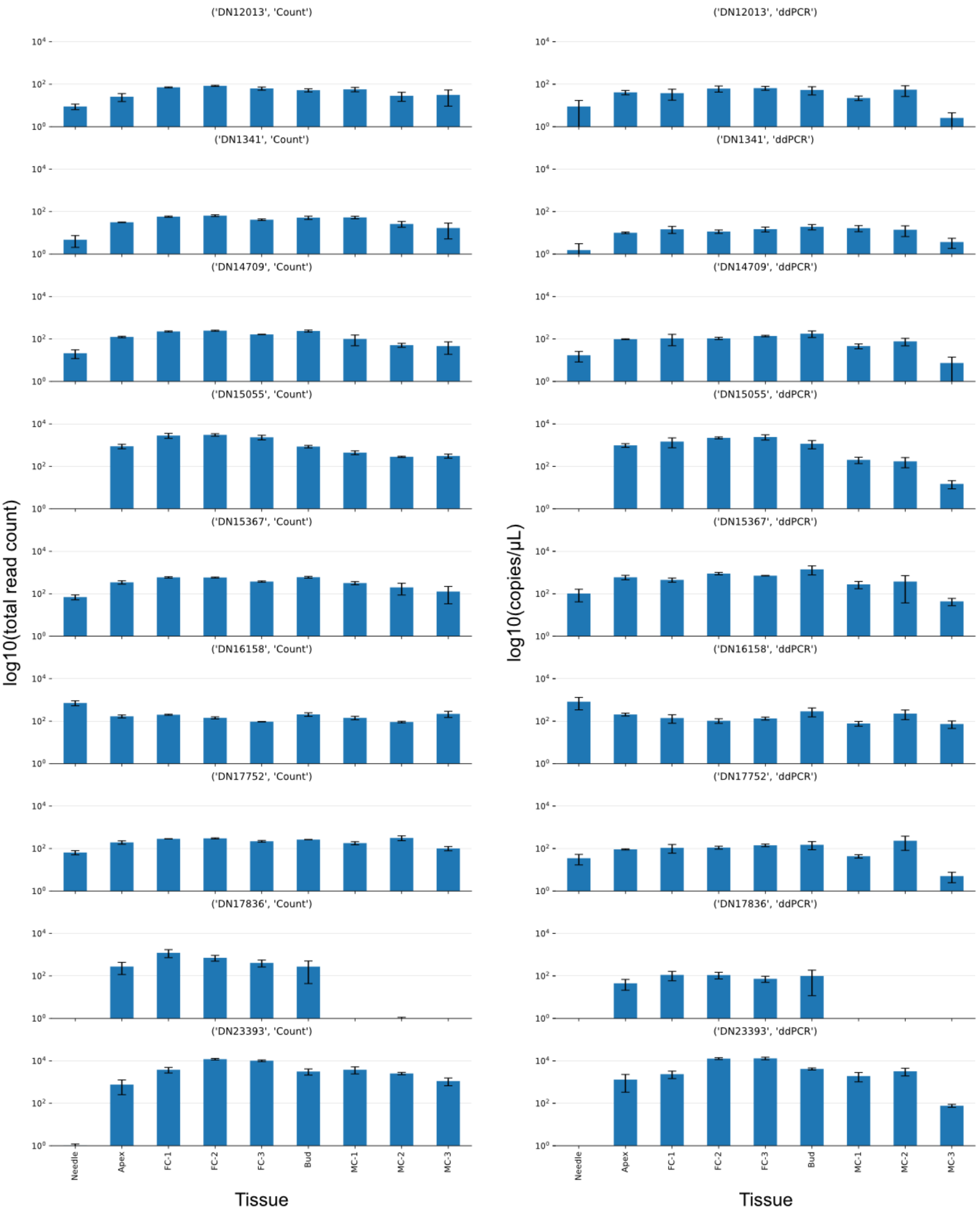


**Figure S3.** Validation of RNA-seq expression levels by ddPCR analysis. Side-by-side expression profile. On the left RNAseq counts compared to ddPCR detection on the right.

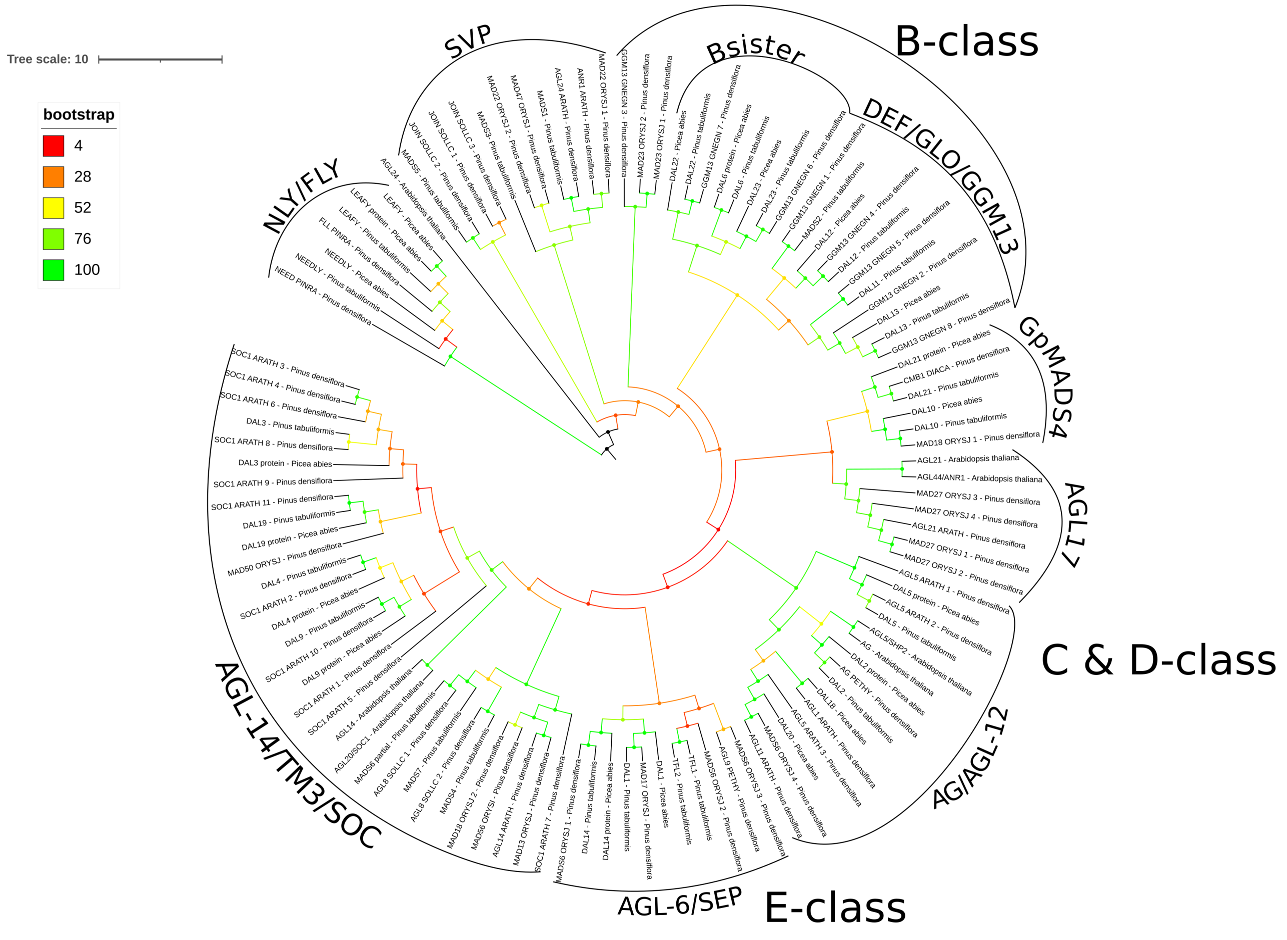


**Figure S4.** Phylogenetic relationships of type II MADS (MIKC-C) box proteins from *P. densiflora, P. tabuliformis, Picea abies* and *A. thaliana*. Protein alignment of the 54 *P. densiflora* genes was performed with T-Coffee multiple sequence alignment package. The maximum likelihood tree was calculated using RAxML - Randomized Axelerated Maximum Likelihood method with 1000 bootstrap replicates. The *NEEDLY/LEAFY* branch was used for rooting the tree. Scale bar: 1 substitutions per site.

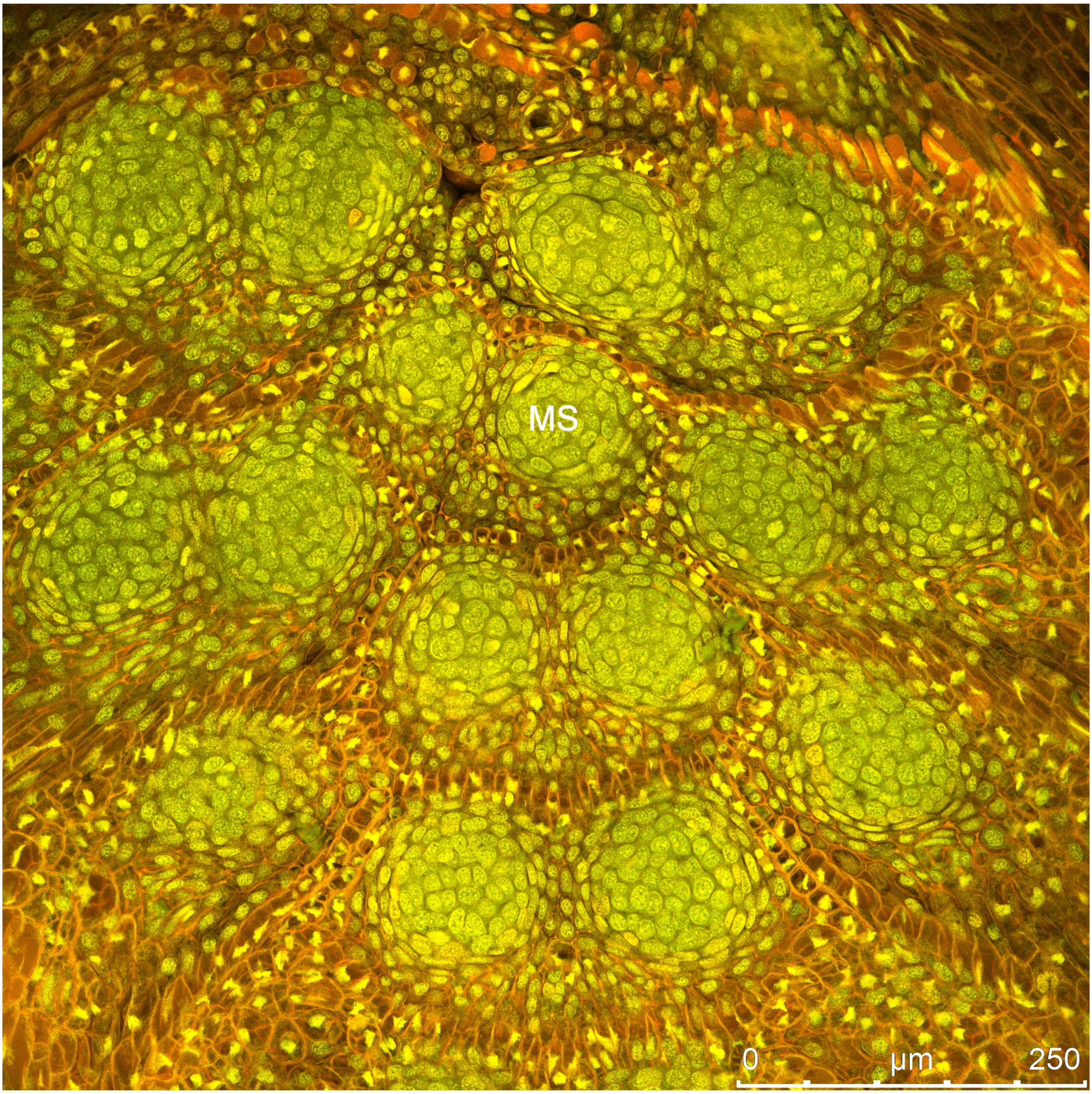


**Figure S5.** Microscopy of ”Bud” tissue evidencing initiation of male cone microsporangia (MS) in some of the sampled material collected at the earliest time points.
